# Systematic review of overlapping microRNA patterns in COVID-19 and idiopathic pulmonary fibrosis

**DOI:** 10.1186/s12931-023-02413-6

**Published:** 2023-04-15

**Authors:** Julien Guiot, Monique Henket, Claire Remacle, Maureen Cambier, Ingrid Struman, Marie Winandy, Catherine Moermans, Edouard Louis, Michel Malaise, Clio Ribbens, Renaud Louis, Makon-Sébastien Njock

**Affiliations:** 1 Laboratory of Pneumology, GIGA Research Center, University of Liège, University Hospital of Liège, Liège, Belgium; 2 Laboratory of Molecular Angiogenesis, GIGA Research Center, University of Liège, Liège, Belgium; 3 Laboratory of Gastroenterology, GIGA Research Center, University of Liège, University Hospital of Liège, Liège, Belgium; 4 Laboratory of Rheumatology, GIGA Research Center, University of Liège, University Hospital of Liège, Liège, Belgium; 5 Fibropole Research Group, University Hospital of Liège, Liège, Belgium

**Keywords:** COVID-19, Idiopathic pulmonary fibrosis, microRNA, Post-COVID-19 lung fibrosis

## Abstract

**Background:**

Pulmonary fibrosis is an emerging complication of SARS-CoV-2 infection. In this study, we speculate that patients with COVID-19 and idiopathic pulmonary fibrosis (IPF) may share aberrant expressed microRNAs (miRNAs) associated to the progression of lung fibrosis.

**Objective:**

To identify miRNAs presenting similar alteration in COVID-19 and IPF, and describe their impact on fibrogenesis.

**Methods:**

A systematic review of the literature published between 2010 and January 2022 (PROSPERO, CRD42022341016) was conducted using the key words (COVID-19 OR SARS-CoV-2) AND (microRNA OR miRNA) or (idiopathic pulmonary fibrosis OR IPF) AND (microRNA OR miRNA) in Title/Abstract.

**Results:**

Of the 1988 references considered, 70 original articles were appropriate for data extraction: 27 studies focused on miRNAs in COVID-19, and 43 on miRNAs in IPF. 34 miRNAs were overlapping in COVID-19 and IPF, 7 miRNAs presenting an upregulation (miR-19a-3p, miR-200c-3p, miR-21-5p, miR-145-5p, miR-199a-5p, miR-23b and miR-424) and 9 miRNAs a downregulation (miR-17-5p, miR-20a-5p, miR-92a-3p, miR-141-3p, miR-16-5p, miR-142-5p, miR-486-5p, miR-708-3p and miR-150-5p).

**Conclusion:**

Several studies reported elevated levels of profibrotic miRNAs in COVID-19 context. In addition, the balance of antifibrotic miRNAs responsible of the modulation of fibrotic processes is impaired in COVID-19. This evidence suggests that the deregulation of fibrotic-related miRNAs participates in the development of fibrotic lesions in the lung of post-COVID-19 patients.

**Supplementary Information:**

The online version contains supplementary material available at 10.1186/s12931-023-02413-6.

## What is already known on this topic

An emerging complications of SARS-CoV-2 infection is pulmonary fibrosis. In this study, we speculate that patients with COVID-19 and idiopathic pulmonary fibrosis (IPF) may share aberrant expressed miRNAs associated to the progression of lung fibrosis.

## What this study adds

This is the first review to identify miRNAs presenting similar alteration in COVID-19 and IPF. Interestingly, these miRNAs are key regulators of fibrosis processes. The deregulation of these fibrotic-related miRNAs may participate in the development of fibrotic lesions in the lung of post-COVID-19 patients.

## How this study might affect research, practice or policy

The study of these miRNAs may help to decipher molecular pathways involved in the development of lung fibrosis in post-COVID-19 patients.

## Introduction

Coronavirus disease 2019 (COVID-19), caused by severe acute respiratory syndrome coronavirus 2 (SARS-CoV-2), has infected more than 523 million persons and caused over 6.3 million deaths worldwide until May 2022 [1]. SARS-CoV-2 primarily affects the lungs, inducing a range of clinical manifestations, from asymptomatic to severe form characterized by acute respiratory distress syndrome (ARDS) and some immune-mediated lung complications, that require intensive care treatment and mechanical ventilation and can ultimately result in respiratory failure and death [2–5].

An emerging complication of SARS-CoV-2 infection is pulmonary fibrosis [6–10]. A recent meta-analysis study by Hama Amin et al. shows that a significant portion of recovered COVID-19 patients (44.9%) appear to have developed pulmonary fibrosis, which may persist over time [10]. The prevalence of post-COVID-19 fibrosis will become more apparent in time, but early analysis from patients with COVID-19 highlighted a high level of fibrotic lung function abnormalities [11–15]. In a recent study, McGroder et al. found that among survivors of severe COVID-19, 20% of non-mechanically ventilated and 72% of mechanically ventilated patients had fibrotic-like radiographic abnormalities 4 months after hospitalization, which correlates with loss of lung function and cough [12, 16]. Similarly, Aul et al. reported that patients who had severe COVID-19 infection, particularly those who were intubated and who have persistent breathlessness are at risk of developing post-COVID-19 pulmonary fibrosis [14]. In a recent study, they showed that up to 9.3% of post-COVID-19 patients with persistent respiratory symptoms present pulmonary fibrosis. In a multicentric observational study including 600 COVID-19 cases with lung involvement, Patil et al. observed lung fibrosis in 13.66% of post-COVID-19 pneumonia patients [15].

Idiopathic pulmonary fibrosis (IPF) is the archetypal progressive fibrosing interstitial lung disease, of unknown etiology and cure which leads to rapid death (2–3 years after diagnosis) [17–21]. IPF is characterized by progressive and irreversible destruction of the lung architecture caused by excessive extracellular matrix (ECM) deposition and remodeling, resulting in the formation of fibrotic scar that ultimately leads to organ destruction and death from respiratory failure [22, 23].microRNAs (miRNAs) are small noncoding RNA molecules (20–22 nucleotides) that post-transcriptionally modulate gene expression by blocking the translation or inducing degradation of target mRNAs [24–28]. Several studies reported the dysregulation of the levels of circulating miRNAs in lung diseases [29, 30]. Previously, we identified a unique signature of three sputum-derived miRNAs presenting an aberrant expression in IPF patients compared to healthy donors [29]. Besides their capacity as potential biomarkers of lung diseases, miRNAs are essential regulators of various cellular processes, including fibrosis [27, 31–33]. Several studies have shown that miRNAs also participate in SARS-CoV-2 infection and pathogenesis through different mechanisms [34, 35], such as: host cell miRNA expression interfering with SARS-CoV-2 cell entry [36, 37]; SARS-CoV-2-derived RNA transcripts acting as competitive endogenous RNAs that may attenuate host cell miRNA expression [38–41]; and host cell miRNA expression modulating SARS-CoV-2 replication [38, 42–44]. In addition, miRNAs have also been implicated in COVID-19 associated manifestations, including pulmonary fibrosis [45].

Because a portion of post-COVID-19 patients develops pulmonary fibrosis, we speculate that COVID-19 and IPF patients share aberrant expressed miRNAs that may be implicated in lung fibrosis. Therefore, the objective of this systematic review was to identify miRNAs presenting similar alterations in COVID-19 and IPF, and to present their impact on fibrogenesis.

## Methods

### Review question

The objective of this systematic review was to identify miRNAs presenting similar alterations in COVID-19 and IPF, and to present their impact on fibrogenesis.

### Data sources and eligibility criteria

**Fig. 1:**
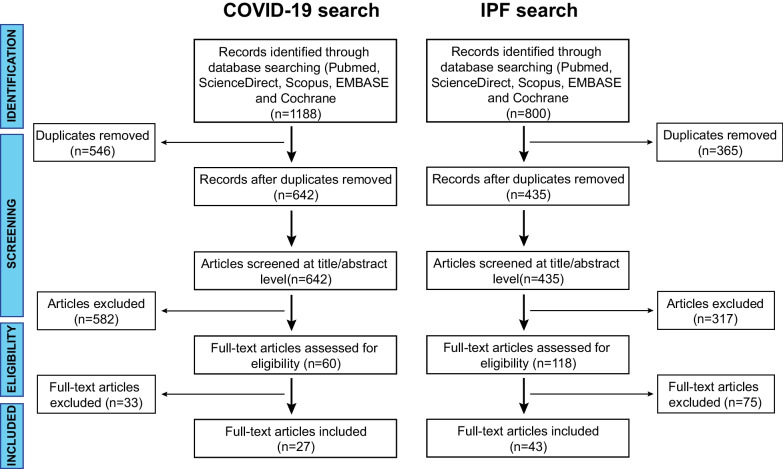
Flow diagram of the systematic research method for detecting matching miRNAs in COVID-19 and IPF

A systematic review of the literature was conducted to search for all articles reporting the deregulation of circulating or cellular miRNAs related to COVID-19 and IPF between 2010 and January 2022 according to PRISMA (Preferred Reporting Items for Systematic Reviews and Meta-Analyses) guidelines [46], with the relative flow diagram shown in Fig. 1. This study satisfied all the recommended items reported in the PRISMA 2020 checklist available (Additional file 1: Fig. S1). The protocol of this synthesis of the current literature has been registered in the International Prospective Register of Systematic Reviews (PROSPERO) database (CRD42022341016). The systematic review was conducted using a defined search strategy by two investigators (JG and MSN) using electronic databases (Pubmed, ScienceDirect, Scopus, EMBASE and Cochrane).

### Inclusion/exclusion criteria

The databases were searched using the keywords (COVID-19 OR SARS-CoV-2) AND (microRNA OR miRNA) or (idiopathic pulmonary fibrosis OR IPF) AND (microRNA OR miRNA) in Title/Abstract. We considered all studies that reported circulating miRNAs in either COVID-19/SARS-CoV-2 and/or IPF in human samples. Studies that are excluded in our meta-analysis met the following criteria: (1) reviews, letters, correspondence, expert opinion, and editorial; (2) animal or in vitro studies; (3) duplicate articles.

### Data collection

Two independent reviewers (JG and MSN) performed the initial screening of study titles and abstracts, based on predefined inclusion and exclusion criteria, as mentioned above. After the selection of potential eligible papers using the title and the abstract, JG and MSN independently retrieved the full-text articles to assess the final eligibility. A third reviewer (MH) resolved disagreements. From a total of 1988 research articles that were obtained after an extensive database search, 70 original studies were selected for data extraction (Fig. 1).

### Outcomes of interest and data extraction

After the selection of eligible papers, JG and MSN extracted the following information independently: name of the first author, year of publication, country of study, methodology (miRNA related and other relevant methods), sample source (biological material (patients’ samples and/or cell lines) and online databases), miRNAs analyzed, main results, and conclusions.

## Results

The COVID-19 search identified 1188 articles and the IPF search 800 articles, for a total of 1988 articles. After the removal of 911 duplicates and 899 through the screening of titles and abstracts, we reviewed 178 full-text articles for eligibility and subsequently excluded 108. In the end, this resulted in a total of 70 articles related to deregulated miRNAs in COVID-19 and IPF: 27 studies focused on miRNAs in COVID-19, and 43 on miRNAs in IPF (Fig. 1).

Subsequent analysis revealed that 147 miRNAs were dysregulated in COVID-19 context (Additional file 2: Table S1), and 113 in IPF (Additional file 3: Table S2). A total of 34 miRNAs were overlapping in COVID-19 and IPF, 7 miRNAs presenting an upregulation in COVID-19 and IPF (miR-19a-3p, miR-200c-3p, miR-21-5p, miR-145-5p, miR-199a-5p, miR-23b, and miR-424) (Table 1), 9 miRNAs presenting a downregulation (miR-17-5p, miR-20a-5p, miR-92a-3p, miR-141-3p, miR-16-5p, miR-142-5p, miR-486-5p, miR-708-3p, and miR-150-5p) (Table 2), and 18 miRNAs presenting an opposite regulation in COVID-19 and IPF (Table 3).

**Table 1:** Overlapping upregulated miRNAs between COVID-19 and IPF

miRNA	Disease	Study	Regulation	Biological material	Subject	Method	Outcome summary
miR-19a-3p	COVID-19	M Fayyad-Kazan et al*.* [47]	**↑**	Plasma	COVID-19 = 6, healthy controls = 6	qPCR array, qPCR	Plasma miR-19a-3p level could serve as potential diagnostic biomarker for SARS-CoV-2-infection
	IPF	T Kadota et al. [48]	**↑**	Lung fibroblast-derived EVs	IPF = 20, healthy controls = 26	qPCR	IPF lung fibroblast-derived EVs contain elevated level of miR-19a-3p
miR-200c-3p	COVID-19	R Pimenta et al. [49]	**↑**	Saliva	COVID-19 = 72, controls = 39	qPCR	miR-200c-3p is a predictor of severity independent of COVID-19 risk factors
		MI Mitchell et al. [50]	**↑**	Serum-derived EVs	COVID-19 patients: severe (n = 17) *vs* mild (n = 13	Small-RNA sequencing, qPCR	miR-200c-3p is upregulated in serum-derived EVs with COVID-19 severity
	IPF	G Yang et al. [51]	**↑**	Serum	Rapidly progressive IPF = 32, slowly progressive IPF = 36, healthy controls = 32	miRNA array, qPCR	Circulating miRNAs in serum (such as miR-200c-3p) could be potentially served as novel regulators influencing disease progression of IPF
miR-21-5p	COVID-19	I Saulle et al. [54]	**↑**	Plasma	COVID-19 = 15, controls = 6	qPCR array	Combination of dysregulated miRNAs and antiviral/immune factors seems to control both the infection and the dysfunctional immune reaction
		A Garg et al. [55]	**↑**	Blood	COVID-19 = 10, healthy controls = 4	Small-RNA sequencing	New insights into inflammation regulatory mechanisms of miRs in COVID-19, which may provide novel diagnostic biomarkers and therapeutic avenues for COVID-19 patients
	IPF	S Sato et al. [110]	**↑**	IPF fibrocytes (BALF), EVs		qPCR	Fibrocytes from BALF collected from fibrotic interstitial pneumonia patients showed higher miR-21-5p expression than those from other patients
		T Makiguchi et al. [58]	**↑**	Serum-derived EVs	IPF = 41, healthy controls = 21	qPCR	EV miR-21-5p as potential prognostic biomarker for IPF
		G Yang et al. [51]	**↑**	Serum	Rapidly progressive IPF = 32, slowly progressive IPF = 36, healthy controls = 32	qPCR array, qPCR	Circulating miRNAs in serum could be potentially served as novel regulators influencing disease progression of IPF
		P Li et al. [56]	**↑**	Serum	IPF = 76, healthy controls = 73	Microarray, qPCR	Altered expression levels of miR-21-5p, miR-155 and miR-101-3p were associated with FVC and radiological features in IPF
		M Yamada et al. [52]	**↑**	Lung, AECs	IPF = 3, healthy controls = 3	qPCR	miR-21-5p is increased in AECs during lung fibrosis and it promotes epithelial-mesenchymal transition
		P Li et al. [57]	**↑**	Serum	IPF = 65, healthy controls = 65	qPCR	Serum miR-21-5p is associated with IPF and the degree of damage indicated by FVC and radiologic examinations
miR-145-5p	COVID-19	A Parray et al. [59]	**↑**	Blood	COVID-19 patients: severe (n = 9) vs asymptomatic (n = 10) severe (n = 9) vs mild (n = 10)	Microarray	Unique miRNA and snoRNA profile that is associated with a higher risk of severity in a cohort of SARS-CoV-2 infected patients
		MI Mitchell et al. [50]	**↑**	Serum-derived EVs, whole serum	COVID-19 patients: severe (n = 17) *vs* mild (n = 13)	Small-RNA sequencing, qPCR	miR-145-5p is upregulated in serum-derived EVs with disease severity
	IPF	T Kadota et al. [48]	**↑**	Lung fibroblast-derived EVs	IPF = 20, healthy controls = 26	Microarray, qPCR	IPF lung fibroblast-derived EVs contain elevated levels of miR-145-5p, miR-23b-3p and miR-494-3p, inducing epithelial—cell senescence by targeting SIRT3, indeed acting as paracrine mediator in the pathogenesis of IPF
miR-199a-5p	COVID-19	D de Gonzalo-Calvo et al. [60]	**↑**	Plasma	COVID-19 patients: ICU (n = 36) *vs* ward (n = 43)	qPCR array	Signature of three miRNAs (miR-148a-3p, miR-451a and miR-486-5p) that distinguishes between ICU and ward patients
	IPF	G Yang et al. [51]	**↑**	Serum	Profiling: Rapidly progressive IPF = 32, slowly progressive IPF = 36, healthy controls = 32	qPCR array, qPCR	Circulating miRNAs in serum could be potentially served as novel regulators influencing disease progression of IPF
		CL Lino Cardenas et al. [61]	**↑**	Lung	IPF = 94, healthy controls = 83	qPCR	MiR-199a-5p behaves as a major mediator of lung fibrosis by promoting the pathogenic activation of pulmonary fibroblasts including proliferation, migration, invasion, and differentiation into myofibroblasts
miR-23b	COVID-19	I Saulle et al. [54]	**↑**	Plasma	COVID-19 = 15, controls = 6	qPCR array	A combination of dysregulated miRNAs and antiviral/immune factors seems to control both the infection and the dysfunctional immune reaction
	IPF	T Kadota et al. [48]	**↑**	Lung fibroblast-derived EVs	IPF = 20, healthy controls = 26	Microarray, qPCR	IPF lung fibroblast-derived EVs contain elevated levels of miR-145-5p, miR-23b and miR-494-3p, inducing epithelial-cell senescence by targeting SIRT3, indeed acting as paracrine mediator in IPF pathogenesis
miR-424	COVID-19	MI Mitchell et al. [50]	**↑**	Serum-derived EVs, Whole serum	COVID-19: severe (n = 17) *vs* mild (n = 13)	qPCR array, qPCR	miR-146a and miR-126-3p are significantly downregulated in serum-derived EVs with disease severity
	IPF	T Kadota et al. [48]	**↑**	Lung fibroblast-derived EVs	IPF = 20, healthy controls = 26	Small-RNA Sequencing, qPCR	IPF lung fibroblast-derived EVs contain elevated levels of miR-424

AECs: alveolar epithelial cells; BALF: Bronchoalveolar lavage fluid; COVID-19: Coronavirus disease 2019; EVs: Extracellular vesicles; FVC: Forced vital capacity; ICU: Intensive care unit; IPF: idiopathic pulmonary fibrosis; qPCR: quantitative PCR; SARS-CoV-2: severe acute respiratory syndrome coronavirus 2. ↑: high levels

**Table 2:** Overlapping downregulated miRNAs between COVID-19 and IPF

miRNA	Disease	Study	Regulation	Biological material	Subject	Method	Outcome summary
miR-17-5p	COVID-19	M Fayyad-Kazan et al*.* [47]	**↓**	Plasma	COVID-19 = 6, healthy controls = 6	qPCR array, qPCR	Decrease of miR-17-5p in the plasma of COVID-19 patients compared to healthy donors
		A Demiray et al. [62]	**↓**	Serum	COVID-19 = 40, healthy controls = 10	qPCR	Decrease of miR-17-5p in the serum of COVID-19 patients compared to healthy donors
	IPF	S Mullenbrock et al. [63]	**↓**	Lung fibroblasts	IPF = 10, healthy controls = 10	Small-RNA sequencing	Decrease of miR-17-5p in fibroblasts of patients with IPF compared to healthy donors
miR-20a-5p	COVID-19	CX Li et al. [64]	**↓**	Blood	COVID-19 = 10, healthy controls = 4	Small-RNA sequencing	New insights into inflammation regulatory mechanisms of miRs in COVID-19, which may provide novel diagnostic biomarkers and therapeutic avenues for COVID-19 patients
	IPF	S Mullenbrock et al. [63]	**↓**	Lung fibroblasts	IPF = 10, healthy controls = 10	Small-RNA sequencing	Decrease of miR-20a-5p in lung fibroblasts of patients with IPF compared to healthy donors
miR-92a-3p	COVID-19	D de Gonzalo-Calvo et al. [60]	**↓**	Plasma	COVID-19 patients: ICU (n = 36) *vs* ward (n = 43)	qPCR array	MiR-92a-3p enable the distinction between ICU and ward patients
	IPF	B Berschneidera et al. [65]	**↓**	Lung, pulmonary fibroblasts	IPF = 8, healthy controls = 7	qPCR	Regulatory role of miR-92a-3p for WNT1-inducible signaling pathway protein 1 expression in pulmonary fibrosis
miR-141-3p	COVID-19	Z Chen et al. [67]	**↓**	PBMCs	COVID-19 = 17, healthy controls = 6	Small-RNA sequencing	miR-141-3p may be biomarkers that predict changes in mild SARS-CoV-2 infection
	IPF	C Huang et al. [69]	**↓**	Lung	IPF = 28 (< 50% FVC *vs* > 80% FVC)	Microarray, qPCR	
miR-16-5p	COVID-19	D de Gonzalo-Calvo et al. [60]	**↓**	Plasma	COVID-19 patients: ICU (n = 36) *vs* ward (n = 43)	qPCR array	Plasma miR-16-5p is differentially expressed between ICU and ward patients
	IPF	D Lacedonia et al. [70]	**↓**	Serum-derived exosomes	IPF = 61, healthy controls = 15	qPCR	Identification of new key players (Let-7d, miR-16-5p) in the pathophysiology of IPF
miR-142-5p	COVID-19	M Fayyad-Kazan et al*.* [47]	**↓**	Plasma	COVID-19 = 6, healthy controls = 6	qPCR array, qPCR	Plasma miR-19a-3p, miR-19b-3p, and miR-92a-3p expression levels could serve as potential diagnostic biomarker for SARS-CoV-2-infection
	IPF	C Huang et al. [69]	**↓**	Lung	IPF = 28 (< 50% FVC *vs* > 80% FVC)	Microarray, qPCR	miR-101 is an antifibrotic miRNA and a potential therapeutic target for pulmonary fibrosis
		P Li et al. [56]	**↓**	Serum	IPF = 76, healthy controls = 73	Microarray, qPCR	Altered expression level of miR-142-5p in IPF context
miR-486-5p	COVID-19	D de Gonzalo-Calvo et al. [60]	**↓**	Plasma	COVID-19 patients: ICU (n = 36) *vs* ward (n = 43)	qPCR array	Signature of three miRNAs (miR-148a-3p, miR-451a and miR-486-5p) that distinguishes between ICU and ward patients
	IPF	X Ji et al. [71]	**↓** **↓**	Lung Serum	IPF = 5, silicosis = 5, healthy controls = 2 silicosis = 60, healthy controls = 20	qPCR	Functional test revealed that miR-486-5p may inhibit pulmonary fibrosis
miR-708-3p	COVID-19	Z Chen et al. [67]	**↓**	PBMCs	COVID-19 = 17, healthy controls = 6	Small-RNA sequencing	miR-340–3p, miR-652–3p, miR-4772–5p, miR-192–5p may be biomarkers that predict changes in mild SARS-CoV-2 infection
	IPF	S Mullenbrock et al. [63]	**↓**	Lung fibroblasts	IPF = 10, healthy controls = 10	Small-RNA sequencing	Over expression of miR-29b-3p, miR-146b-5p, or miR-138-5p decreased expression of distinct sets of fibrotic signature genes
		B Liu et al. [72]	**↓**	PBMCs	IPF = 78, healthy controls = 78	qPCR	Downregulation of miR-708-3p aggravates IPF, and miR-708-3p can serve as a potential therapeutic target for IPF
miR-150-5p	COVID-19	D de Gonzalo-Calvo et al. [60]	**↓**	Plasma	COVID-19 patients: ICU (n = 36) *vs* ward (n = 43)	qPCR array	Signature of three miRNAs (miR-148a-3p, miR-451a and miR-486-5p) that distinguishes between ICU and ward patients
	IPF	NG Casanova et al. [73]	**↓**	PBMCs	IPF = 70 (according to disease severity)	qPCR array	miRNA-driven peripheral blood molecular signatures as valuable and novel biomarkers associated to individuals at high survival risk and for potentially facilitating individualized therapies in IPF disease

AECs: alveolar epithelial cells; BALF: Bronchoalveolar lavage fluid; COVID-19: Coronavirus disease 2019; EVs: Extracellular vesicles; FVC: Forced vital capacity; ICU: Intensive care unit; IPF: idiopathic pulmonary fibrosis; qPCR: quantitative PCR; SARS-CoV-2: severe acute respiratory syndrome coronavirus 2. ↓: low levels

**Table 3:** Overlapping miRNAs with opposite regulation between COVID-19 and IPF

miRNA	Disease	Study	Regulation	Biological material	Subject	Method	Outcome summary
miR-142-3p	COVID-19	Z Chen et al. [67]	**↓**	PBMCs	COVID-19 = 17, healthy controls = 6	Small-RNA sequencing	miR-340–3p, miR-652–3p, miR-4772–5p, miR-192–5p may be biomarkers that predict changes in mild SARS-CoV-2 infection. Some molecules, including hsa-miR-1291, were considered potential targets to predict the emergence of severe symptoms in SARS-CoV-2 infection
		H Tang et al. [77]	**↓**	Whole blood	COVID-19: severe (n = 6) *vs* moderate (n = 6)	Small-RNA sequencing	miR-146a-5p, miR-21-5p, miR-142-3p, and miR-15b-5p are potential contributors to the disease pathogenesis, possibly serving as biomarkers of severe COVID-19
	IPF	J Guiot et al. [33]	**↑** **↑**	Sputum-derived exosomes Plasma-derived exosomes	IPF = 19, healthy controls = 23 IPF = 14, healthy controls = 14	qPCR	Macrophage-derived exosomes may fight against pulmonary fibrosis progression via the delivery of antifibrotic miR-142–3 p to alveolar epithelial cells and lung fibroblasts
		M-S Njock et al. [29]	**↑**	Sputum-derived exosomes	IPF = 16, healthy controls = 14	miRNA qPCR array	First characterisation of miRNA content of sputum-derived exosomes in IPF that identified promising biomarkers for diagnosis and disease severity
miR-15a-5p	COVID-19	M Fayyad-Kazan et al*.* [47]	**↑**	Plasma	COVID-19 = 6, healthy controls = 6	qPCR array, qPCR	Plasma miR-19a-3p, miR-19b-3p, and miR-92a-3p expression levels could serve as potential diagnostic biomarker for SARS-CoV-2-infection
		MI Mitchell et al. [50]	**↑**	Serum-derived EVs, whole serum	COVID-19 patients: severe (n = 17) *vs* mild (n = 13)	Small-RNA sequencing, qPCR	miR-146a and miR-126-3p are significantly downregulated in serum-derived EVs with disease severity
	IPF	Y Chen et al. [74]	**↓**	Lung tissue	IPF = 106, healthy controls = 50	Microarray	miR-15a inhibits fibrogenesis in lung fibroblast and abrogated BLM-induced lung fibrosis in mice. Novel strategies for the prevention and treatment of lung fibrosis
miR-31-5p	COVID-19	RJ Farr et al. [75]	**↑**	Plasma	COVID-19 = 10, healthy controls = 10	Small-RNA sequencing, qPCR	miRNA signature, consisting of miR423-5p, miR-23a-3p, miR-195-5p, could independently classify COVID-19 patients from healthy controls
	IPF	NG Casanova et al. [73]	**↓**	PBMCs	IPF = 70 (according to disease severity)	miRNA qPCR array	miRNA-driven peripheral blood molecular signatures as valuable and novel biomarkers associated to individuals at high survival risk and for potentially facilitating individualized therapies in IPF disease
miR-93-5p	COVID-19	A Demiray et al. [62]	**↓**	Serum	COVID-19 = 40, healthy controls = 10	qPCR	The increase in miR-190a level may be a prognostic factor related to the COVID-19 disease
	IPF	S Mullenbrock et al. [63]	**↑**	Lung fibroblasts	IPF = 10, healthy controls = 10	Small-RNA sequencing	Over expression of miR-29b-3p, miR-146b-5p, or miR-138-5p decreased expression of distinct sets of fibrotic signature genes
miR-96-5p	COVID-19	CX Li et al. [64]	**↓**	Blood	COVID-19 = 10, healthy controls = 4	Small-RNA sequencing	New insights into inflammation regulatory mechanisms of miRs in COVID-19, which may provide novel diagnostic biomarkers and therapeutic avenues for COVID-19 patients
	IPF	RS Nho et al. [78]	**↑**	Lung, pulmonary fibroblasts	IPF = 8, healthy controls = 8	qPCR	The alteration of miR-96 expression in IPF fibroblasts contributes to maintain their pathological phenotype, which may contribute to the progression of IPF
miR-144-3p	COVID-19	CX Li et al. [64]	**↓**	Blood	COVID-19 = 10, healthy controls = 4	Small-RNA sequencing	New insights into inflammation regulatory mechanisms of miRs in COVID-19, which may provide novel diagnostic biomarkers and therapeutic avenues for COVID-19 patients
	IPF	NG Casanova et al. [73]	**↑**	PBMCs	IPF = 70 (according to disease severity)	miRNA qPCR array	miRNA-driven peripheral blood molecular signatures as valuable and novel biomarkers associated to individuals at high survival risk and for potentially facilitating individualized therapies in IPF disease
miR-223	COVID-19	I Saulle et al. [54]	**↑**	Plasma	COVID-19 = 15, controls = 6	qPCR array	A combination of dysregulated miRNAs and antiviral/immune factors seems to control both the infection and the dysfunctional immune reaction
		A Demiray et al. [62]	**↓**	Serum	COVID-19 = 40, healthy controls = 10	qPCR	The increase in miR-190a level may be a prognostic factor related to the COVID-19 disease
	IPF	NG Casanova et al. [73]	**↑**	PBMCs	IPF = 70 (according to disease severity)	MiRNA qPCR array	miRNA-driven peripheral blood molecular signatures as valuable and novel biomarkers associated to individuals at high survival risk and for potentially facilitating individualized therapies in IPF disease
miR-34b	COVID-19	A Demiray et al. [62]	**↓**	Serum	COVID-19 = 40, healthy controls = 10	qPCR	Decrease of miR-34b level in COVID-19 disease
	IPF	S Disayabutr et al. [79]	**↑**	AECs	IPF = 15, healthy controls = 15	miRNA arrays, qPCR	The relative levels of senescence-associated miRNAs miR-34a, miR-34b, and miR-34c were significantly higher in AECs from IPF patients
miR-34c	COVID-19	Z Chen et al. [67]	**↓**	PBMCs	COVID-19 = 17, healthy controls = 6	Small-RNA sequencing	miR-340–3p, miR-652–3p, miR-4772–5p, miR-192–5p may be biomarkers that predict changes in mild SARS-CoV-2 infection
	IPF	S Disayabutr et al. [79]	**↑**	AECs	IPF = 15, healthy controls = 15	miRNA arrays, qPCR	The relative levels of senescence-associated miRNAs miR-34a, miR-34b, and miR-34c were significantly higher in AECs from IPF patients
miR-27a-3p	COVID-19	Z Chen et al. [67]	**↓**	PBMCs	COVID-19 = 17, healthy controls = 6	Small-RNA sequencing	miR-340–3p, miR-652–3p, miR-4772–5p, miR-192–5p may be biomarkers that predict changes in mild SARS-CoV-2 infection. Some molecules, including hsa-miR-1291, were considered potential targets to predict the emergence of severe symptoms in SARS-CoV-2 infection
		D de Gonzalo-Calvo et al. [60]	**↑**	Plasma	COVID-19 patients: ICU (n = 36) *vs* ward (n = 43)	qPCR array	Signature of three miRNAs (miR-148a-3p, miR-451a and miR-486-5p) that distinguishes between ICU and ward patients
	IPF	H Cui et al. [111]	**↓**	Lung fibroblasts (control) and myofibroblasts (IPF)	IPF = 6, healthy controls = 6	qPCR	This study discovered that miR-27a-3p was a negative regulator of lung myofibroblast differentiation and pulmonary fibrosis
miR-29c-3p	COVID-19	I Saulle et al. [54]	**↑** **↑**	Plasma Placenta	COVID-19 = 15, controls = 6	qPCR array	A combination of dysregulated miRNAs and antiviral/immune factors seems to control both the infection and the dysfunctional immune reaction
	IPF	T Xie et al. [76]	**↓**	Alveolar epithelial cells (AECs)	IPF = 7, healthy controls = 4	qPCR	miR-29c maintains epithelial integrity and promotes recovery from lung injury, thereby attenuating lung fibrosis in mice
miR-29a-3p	COVID-19	C Grehl et al*.* [81]	**↓**	Plasma	COVID-19 patients: severe (n = 5) *vs* mild (n = 3)	Small-RNA sequencing	Several of these miRNAs are associated with JAK-STAT response and cytokine storm
		I Saulle et al. [54]	**↑**	Plasma	COVID-19 = 15, controls = 6	qPCR array	A combination of dysregulated miRNAs and antiviral/immune factors seems to control both the infection and the dysfunctional immune reaction
		R Keikha et al. [82]	**↓**	Serum	COVID-19 patients with grade 1 (n = 21), grade 2 (n = 20), grade 3 (n = 20), grade 4 (n = 21), and grade 5 (n = 21)	qPCR	Relative expression of miR-31-3p, miR-29a-3p, and miR-126-3p was down-regulated and relative expression of miR-17-3p was up-regulated with the increase of COVID-19 grade
		T Donyavi et al. [80]	**↑**	PBMCs	COVID-19 = 18, healthy controls = 15	qPCR	miR-29a-3p, miR-155-5p and miR-146a-3p may serve as the novel biomarker for COVID-19 diagnosis
	IPF	E Tsitoura et al. [83]	**↓**	BAL cells	IPF = 45, healthy controls = 17	qPCR	Novel evidence of the involvement of the miR-185/AKT pathway in IPF BAL cells, and support for the use of miR-29a and miR-185 as BAL IPF biomarkers
miR-192–5p	COVID-19	Z Chen et al. [67]	**↓**	PBMCs	COVID-19 = 17, healthy controls = 6	Small-RNA sequencing	miR-340–3p, miR-652–3p, miR-4772–5p, miR-192–5p may be biomarkers that predict changes in mild SARS-CoV-2 infection. Some molecules, including hsa-miR-1291, were considered potential targets to predict the emergence of severe symptoms in SARS-CoV-2 infection
	IPF	M-S Njock et al. [29]	**↑**	Sputum-derived exosomes	IPF = 16, healthy controls = 14	miRNA qPCR array	First characterisation of miRNA content of sputum-derived exosomes in IPF that identified promising biomarkers for diagnosis and disease severity
miR-195-5p	COVID-19	RJ Farr et al. [75]	**↑**	Plasma	COVID-19 = 10, healthy controls = 10	Small-RNA sequencing, qPCR	miRNA signature, consisting of miR423-5p, miR-23a-3p, miR-195-5p, could independently classify COVID-19 patients from healthy controls (99.9% accuracy)
	IPF	C Huang et al. [69]	**↓**	Lung	IPF = 28 (< 50% FVC *vs* > 80% FVC)	microarray, qPCR	miR-101 is an antifibrotic microRNA and a potential therapeutic target for pulmonary fibrosis
miR-1275	COVID-19	RJ Farr et al. [75]	**↓**	Plasma	COVID-19 = 10, healthy controls = 10	Small-RNA sequencing, qPCR	miRNA signature, consisting of miR423-5p, miR-23a-3p, miR-195-5p, could independently classify COVID-19 patients from healthy controls (99.9% accuracy)
	IPF	NG Casanova et al. [73]	**↑**	PBMCs	IPF = 70 (according to disease severity)	miRNA qPCR array	miRNA-driven peripheral blood molecular signatures as valuable and novel biomarkers associated to individuals at high survival risk and for potentially facilitating individualized therapies in IPF disease
miR-27b-3p	COVID-19	D de Gonzalo-Calvo et al. [60]	**↑**	Plasma	COVID-19 patients: ICU (n = 36) *vs* ward (n = 43)	qPCR array	Signature of three miRNAs (miR-148a-3p, miR-451a and miR-486-5p) that distinguishes between ICU and ward patients
	IPF	C Huang et al. [69]	**↓**	Lung	IPF = 28 (< 50% FVC *vs* > 80% FVC)	Microarray, qPCR	miR-101 is an antifibrotic microRNA and a potential therapeutic target for pulmonary fibrosis
miR-15b-5p	COVID-19	H Tang et al. [77]	**↑**	Whole blood	COVID-19 patients: severe (n = 6) *vs* moderate (n = 6)	Small-RNA sequencing	miR-146a-5p, miR-21-5p, miR-142-3p, and miR-15b are potential contributors to the disease pathogenesis, possibly serving as biomarkers of severe COVID-19
	IPF	Y Chen et al. [74]	**↓**	Lung tissue	IPF = 106, healthy controls = 50	Microarray	miR-15a-5p inhibits fibrogenesis in lung fibroblast and abrogated BLM-induced lung fibrosis in mice
miR-190a-5p	COVID-19	A Demiray et al. [62]	**↑**	Serum	COVID-19 = 40, healthy controls = 10	qPCR	The increase in miR-190a level may be a prognostic factor related to the COVID-19 disease
	IPF	S Mullenbrock et al. [63]	**↓**	Lung fibroblasts	IPF = 10, healthy controls = 10	Small-RNA sequencing	Over expression of miR-29b-3p, miR-146b-5p, or miR-138-5p decreased expression of distinct sets of fibrotic signature genes

AECs: alveolar epithelial cells; BALF: Bronchoalveolar lavage fluid; COVID-19: Coronavirus disease 2019; EVs: Extracellular vesicles; FVC: Forced vital capacity; ICU: Intensive care unit; IPF: idiopathic pulmonary fibrosis; qPCR: quantitative PCR; SARS-CoV-2: severe acute respiratory syndrome coronavirus 2. ↑: high levels, ↓: low levels

### Overlapping miRNAs between COVID-19 and IPF: upregulated miRNAs

#### MiR-19a-3p in COVID-19 and IPF

M Fayyad-Kazan et al*.* have shown that eight miRNAs were differentially expressed in the plasma of COVID-19 patients versus healthy donors, among which miR-19a-3p being up-regulated whilst miR-17-5p being down-regulated in SARS-CoV-2-infected patients [47]. Similarly, Kadota et al*.* have shown that miR-19a-3p is upregulated in extracellular vesicles (EVs) derived from lung fibroblasts of IPF patients [48].

#### MiR-200c-3p in COVID-19 and IPF

MiR-200c-3p expression is upregulated in saliva [49] and serum [50] of COVID-19 patients, as well as serum of IPF patients [51]. Controversial results have been observed in the lung of IPF patients, with decreased expression of miR-200c-3p [52, 53].

#### MiR-21-5p in COVID-19 and IPF

The data collected on miR-21-5p demonstrate that its expression is upregulated in plasma [54] and serum [55] of COVID-19 patients, as well as in serum [51, 56, 57], serum-derived EVs [58] and alveolar epithelial cells (AECs) [52] of IPF patients compared to healthy controls.

#### MiR-145-5p in COVID-19 and IPF

MiR-145-5p is upregulated in COVID-19 patients [50, 59], particularly in EVs derived from the blood of patients with COVID-19 [50]. Similarly, this miRNA is upregulated in EVs generated in the IPF context [48]. Indeed, Kadota et al. found that lung fibroblast-derived EVs from IPF patients (n = 20) contain an elevated level of miR-145-5p compared to controls without IPF (n = 26) [48].

#### MiR-199a-5p in COVID-19 and IPF

MiR-199a-5p is upregulated in the blood of COVID-19 [60] and IPF patients [51] compared to healthy donors. In addition, Lino Cardenas et al. have shown that miR-199a-5p pulmonary expression was significantly increased in IPF patients [61].

#### MiR-23b and miR-424 in COVID-19 and IPF

MiR-23b is upregulated in plasma of COVID-19 patients [54] as well as in EVs derived from lung fibroblasts of IPF patients [48]. Similarly, the level of miR-424 is high in EVs from serum of COVID-19 [50] and lung fibroblasts of IPF patients [48].

### Overlapping miRNAs between COVID-19 and IPF: downregulated miRNAs

#### MiR-17–92 cluster members in COVID-19 and IPF

M Fayyad-Kazan et al*.* have shown that miR-17-5p is down-regulated in SARS-CoV-2-infected patients [47]. The modulation of miR-17-5p in COVID-19 context was confirmed in another study by Demiray et al*.* Indeed, they highlighted the decrease of miR-17-5p in the serum of COVID-19 patients compared to healthy donors [62]. Similarly, miR-17-5p is downregulated in lung fibroblasts of IPF patients [63].

In addition, two other members of miR-17–92 cluster, miR-20a-5p and miR-92a-3p, are downregulated in both COVID-19 [60, 64] and IPF patients [63, 65].

#### miR-141-3p in COVID-19 and IPF

miR-141-3p is a member of the miR-200 family that has been associated with the regulation of the epithelial-mesenchymal transition (EMT) phenotype [66]. This miRNA is downregulated in COVID-19 peripheral blood mononuclear cells (PBMCs) [67] and IPF patients’ lungs [68, 69].

#### miR-16-5p in COVID-19 and IPF

miR-16-5p is downregulated in these two diseases [60, 70]. In a recent study, Gonzalo-Calvo et al. examined the plasma miRNA profile of hospitalized COVID-19 patients (n = 36) and identified several miRNAs dysregulated in ICU patients compared to ward patients (n = 43), among which miR-16-5p (downregulated) [60]. Lacedonia et al. also reported a reduction of the levels of miR-16-5p in the serum of a group of IPF patients (n = 61) compared to healthy controls (n = 15) [70].

#### miR-142-5p in COVID-19 and IPF

A study comparing patients affected by COVID-19 (n = 6) to healthy volunteers (n = 6) reported that the level of miR-142-5p being down-regulated [47]. In another study by Li et al. [64], whole-transcriptomic sequencing of blood samples from COVID-19 patients (n = 10) and healthy donors (n = 4) enabled to identified 23 differentially expressed miRNAs, among which an upregulation of miR-142-5p. In the IPF context, several studies reported a reduced level of miR-142-5p in serum [56] and lung tissues [69] of IPF patients compared to healthy controls.

#### miR-486-5p in COVID-19 and IPF

In the COVID-19 context, D de Gonzalo-Calvo et al. identified a signature of three miRNAs, among which miR-486-5p, that distinguishes between ICU and ward patients [60]. Similarly, miR-486-5p expression was decreased in serum samples from patients with silicosis, as well as the lung tissues of patients with either silicosis or IPF, compared with healthy donors [71].

#### MiR-708-3p in COVID-19 and IPF

miR-708-3p is downregulated in the PBMCs of COVID-19 [67] and IPF patients [63, 72] compared to healthy controls. In a recent study, Chen et al. have shown that the expression level of miR-708-3p is reduced between COVID-19 patients with mild and serious symptoms, and between COVID-19 patients and healthy controls [67]. Another study by Liu et al. showed that miR-708-3p expression was lower in the PBMCs of the patients with IPF than in those of the normal individuals [72].

#### MiR-150-5p in COVID-19 and IPF

MiR-150-5p is downregulated in the plasma of COVID-19 patients. Indeed, a study by D de Gonzalo-Calvo et al. reported a decrease of the level of miR-150-5p in critically ill COVID-19 patients [60]. Similarly, there is a reduction of the expression of miR-150-5p in the PBMCs of IPF patients according to disease severity [73].

### Overlapping miRNAs between COVID-19 and IPF: miRNAs with opposite regulation

Several other overlapping miRNAs are of potential interest, but they present an opposite regulation in COVID-19 and IPF context (Table 3). Indeed, opposite regulation have been reported for miR-15a-5p [47, 50, 74], miR-31-5p [73, 75], miR-29c-3p [54, 76], miR-195-5p [69, 75], miR-27b-3p [60, 69], miR-15b-5p [74, 77] and miR-190a-5p [62, 63]: both are upregulated in COVID-19 and downregulated in IPF. For example, miR-15a-5p and miR-15b-5p are upregulated in blood of COVID-19 patients [47, 77], as well as in EVs derived from serum of COVID-19 patients for miR-15a-5p [50]. In contrast, the levels of these two miRNAs are decreased in the lung of IPF patients compared to healthy controls [74].

On the other hand, miR-142-3p [29, 33, 67, 77], miR-93-5p [62, 63], miR-96-5p [64, 78], miR-144-3p [64, 73], miR-34b [62, 79], miR-34c [67, 79], miR-192-5p [29, 67], and miR-1275 [73, 75] present a downregulation in COVID-19 and an upregulation in IPF (Table 3). Two studies reported a downregulation of miR-142-3p in blood [77] and PBMCs [67] of COVID-19 patients compared to controls, whereas two others showed an upregulation of miR-142-3p in exosomes derived from sputum [29] and plasma [33] of IPF patients compared to healthy controls. Njock et al. characterized for the first time the miRNA content of exosomes from the sputum of patients with IPF (n = 16) compared to healthy controls (n = 14) and identified a unique signature of three altered miRNAs: miR-142-3p, miR-33a-5p and let-7d-5p [29]. Interestingly, they found a negative correlation between miR-142-3p and diffusing capacity of the lungs for carbon monoxide/alveolar volume.

Other miRNAs present a controversial regulation in COVID-19 (Table 3). Two studies reported the upregulation of miR-29a-3p in the plasma [54] and PBMCs [80] of COVID-19 patients compared to healthy subjects, whereas two others reported its downregulation in the plasma [81] and serum [82] of COVID-19 patients. In IPF context, a study by Tsitoura et al. reported the reduction of the level of miR-29a-3p in BAL cells [83].

## Discussion

**Fig. 2:**
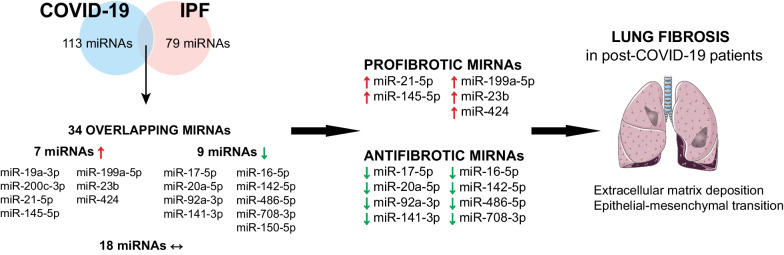
Overlapping of dysregulated miRNAs in COVID-19 and IPF: impact in the development of fibrotic lesions in the lung of post-COVID-19 patients

It is well established that miRNAs are essential regulators of pulmonary fibrosis, by targeting several processes including ECM deposition and EMT. The miRNA balance which participates in the maintenance of physiological state in the lung is disrupted during IPF, participating in the progression of pulmonary fibrosis [84]. Similarly, we hypothesize that the miRNAs dysregulated in COVID-19 participate in the apparition of lung fibrogenesis by inducing collagen deposition and myofibroblast transformation. For this, we identified miRNAs similarly expressed in COVID-19 and IPF (Tables 1 and 2), and reported their implication in the pathogenesis of IPF (Fig. 2).

### Upregulated miRNAs during COVID-19: impact on fibrotic processes

The miR-200 family consisting of 5 members (miR-200a, miR-200b, miR-200c-3p, miR-141-3p and miR-429) has been shown to play crucial roles in the regulation of pulmonary fibrosis and is potentially important for the diagnosis and treatment of IPF [53]. Two members of the miR-200 family are dysregulated similarly in COVID-19 and IPF: miR-200c-3p (upregulated) and miR-141-3p (downregulated). Yang et al. have shown that miR-200 family members can reverse the fibrogenic activity of pulmonary fibroblasts from both bleomycin-treated mice and IPF patients [53]. Indeed, they have demonstrated that the introduction of miR-200c-3p diminishes bleomycin-induced pulmonary fibrosis in mice, suggesting that restoring miR-200c-3p may be a novel approach for treating lung fibrosis. In addition, the miR-200 family is also able to regulate the progression of pulmonary fibrosis by suppressing EMT of alveolar epithelial cells (AECs) [85].

Several studies demonstrated the profibrotic impact of miR-21-5p. Previously, Liu et al. have found an upregulation of miR-21-5p in the lungs of mice with bleomycin-induced fibrosis and also in the lungs of patients with IPF, primarily localized in myofibroblasts [86]. The overexpression of miR-21-5p in pulmonary fibroblasts increased the expression of profibrotic markers, such as fibronectin (FN) and α-smooth muscle actin (α-SMA) [86]. The authors have demonstrated that miR-21-5p induces fibrosis by interfering with SMAD7, a modulator of fibrotic pathway. MiR-21-5p inhibition suppressed morphological markers of pulmonary fibrosis in a mouse model of IPF and inversely regulates TGF-β1-induced ECM protein expression in human pulmonary fibroblast cell lines [87]. Another study by Yamada et al. has shown that miR-21-5p also facilitates EMT, one of the major processes underlying the dissemination of fibrotic injury [52].

Studies focusing on the functional properties of miR-145-5p in IPF context have been performed and highlighted its profibrotic property. Indeed, the overexpression of miR-145-5p in lung fibroblasts increased SMA-α expression, enhanced contractility, and promoted the formation of focal and fibrillar adhesions, and the depletion of miR-145-5p is protective against bleomycin-induced lung fibrosis [88]. It has been shown that miR-145-5p is also implicated in the induction of EMT process [89].

Lino Cardenas et al. have shown that miR-199a-5p pulmonary expression was significantly increased in IPF patients, and demonstrated that this miRNA behaves as a major mediator of lung fibrosis by promoting the pathogenic activation of pulmonary fibroblasts including proliferation, migration, invasion, and differentiation into myofibroblasts [61]. It has been shown that miR-199a-5p is also increased during liver fibrosis and that miR-199a-5p plays a role in hepatic stellate cell activation, promoting α-SMA production and fibrosis progression [90].

Kadota et al. highlighted that the expression of miR-23b and miR-424 are elevated in EVs derived from IPF lung fibroblasts compared to that in healthy controls [48]. These EVs are able to induce epithelial-cell senescence by targeting SIRT3, indeed acting as paracrine mediator in IPF pathogenesis. In addition, miR-424 induces the myofibroblast differentiation during EMT by potentiating the TGF-β signaling pathway [91, 92].

### Downregulated miRNAs during COVID-19: impact on fibrotic processes

The polycistronic miR-17–92 cluster encodes six individual miRNAs: miR-17-5p, miR-18a-5p, miR-19a-3p, miR-19b-3p, miR-20a-5p and miR-92a-3p [93, 94]. Interestingly, three of them are downregulated in COVID-19 and IPF: miR-17-5p, miR-20a-5p, and miR-92a-3p (Table 2). Dakhlallah et al. have reported the reduction of miR-17∼92 cluster expression in lung tissue from IPF patients, and its re-expression leads to reduced fibrotic gene expression in vitro and in vivo [95]. In addition, several studies have shown that miR-17∼92 cluster members modulate the expression of matrix metalloproteinases implicated in IPF [96–98]. All these studies clearly demonstrated that the deregulation of miR-17∼92 cluster members are implicated in IPF development, and may participate in the progression of pulmonary fibrosis observed in post-COVID patients.

MiR-141-3p is a member of the miR-200 family that has been associated with the regulation of EMT phenotype [66]. A study by Huang et al. has shown that upregulation of miR-141-3p in tubular epithelial hindered EMT by enhancing E-cadherin and decreasing vimentin and fibroblast-specific protein 1 expression [99]. In another study, Qian et al. have shown that miR-141-3p inhibited EMT by targeting Zinc-finger E-box binding homeobox 1 (ZEB1) [68]. The decrease of miR-141-3p observed in COVID-19 patients could participate in the development of fibrotic lesions in the lung.

MiR-16-5p plays an important role in the regulation of EMT [100, 101]. The loss of expression of miR-16-5p observed during transdifferentiation of hepatic stellate cells (HSC) is correlated to the myofibroblast-specific phenotype [102]. The upregulation of miR-16-5p abrogates characteristic functions of myofibroblasts, including collagen and α-SMA expression, reversing myofibroblast phenotype to HSC-like cells [103, 104]. Interestingly, The overexpression of miR-16-5p in exosomes significantly suppressed the enhancing effects of TGF-β1 on proliferation, migration, and collagen (COL1A1) expression of fibroblasts, and attenuated bleomycin-induced skin fibrosis [105]. In an elegant study, Inomata et al. reported the antifibrotic properties of miR-16-5p and demonstrated that these effects occur via the mTORC2 pathway [101].miR-142-5p plays a pivotal role in tissue fibrogenesis, by regulating the switch of macrophage to profibrotic M2 phenotype. Indeed, the inhibition of miR-142-5p in vivo reduces bleomycin-induced lung fibrosis by modulating the polarization of macrophages to M2 phenotype and subsequent profibrotic activation [106].

MiR-486-5p and miR-708-3p also present antifibrotic properties. A study by Ji et al. revealed that the overexpression of miR-486-5p significantly decreased both the distribution and severity of lung lesions in silica-induced mouse model of pulmonary fibrosis compared to control group [71]. Another study by Liu et al. showed that the level of miR-708-3p decreased during fibrosis and inversely correlated with IPF [72]. Therefore, the decrease of these miRNAs might represent a primary pathogenic mechanism underlying the development of lung fibrosis in post-COVID-19 patients.

Interestingly, several downregulated miRNAs in COVID-19 context, but not in IPF, also possess antifibrotic properties, such as miR-142-3p [33] or miR-34c [107]. Guiot et al. have shown that the overexpression of miR-142-3p in alveolar epithelial cells and lung fibroblasts was able to reduce the expression of transforming growth factor β receptor 1 (TGFβ-R1) and profibrotic genes [33]. Furthermore, exosomes isolated from macrophages present antifibrotic properties due in part to the repression of TGFβ-R1 by miR-142-3p transfer in target cells. Following this, overexpression of miR-142-3p represses the expression of profibrotic genes in cardiomyocytes [108] and hepatic stellate cells [109].

### Limitations

The main limitation is the heterogeneity of the individual studies. The differences can be biological, experimental, or variations in technique. For example, the difference in techniques, tissues and biofluids (blood/saliva, lung cells/exosomes), laboratory methods (RT-qPCR, sequencing) and various miRNA profiling platforms in selected studies. The effect of this limitation was reduced by collecting the results of these heterogeneous studies, evaluating their similarities and finding common differentially expressed miRNAs.

## Conclusion

Several studies reported elevated levels of profibrotic miRNAs (miR-21-5p, miR-145-5p, miR-199a-5p, miR-23b and miR-424) in COVID-19 context. In addition, the balance of antifibrotic miRNAs responsible of the modulation of fibrotic processes (miR-17∼92 cluster members (miR-17-5p, miR-20a-5p, miR-92a-3p), miR-141-3p, miR-16-5p, miR-142-5p, miR-486-5p, miR-708-3p) is completely broken in COVID-19 (Fig. 2). All these evidences suggest that the deregulation of fibrotic-related miRNAs (upregulation of profibrotic miRNAs and downregulation of antifibrotic miRNAs) may participate in the development of fibrotic lesions in the lung of post-COVID-19 patients.

## Supplementary Information

**Additional file 1: Figure S1.** PRISMA 2020 checklist

**Additional file 2: Table S1.** miRNA patterns in COVID-19

**Additional file 3: Table S2.** miRNA patterns in IPF

## Data Availability

The protocol of this synthesis of the current literature has been registered in the International Prospective Register of Systematic Reviews (PROSPERO) database (CRD42022341016). The data generated in this study may be available upon reasonable request from the corresponding author.

## Abbreviations

ARDS: Acute respiratory distress syndrome
BALF: Bronchoalveolar lavage fluid
BLM: Bleomycin
COVID-19: Coronavirus disease 2019
ddPCR: Droplet digital PCR
ECM: Extracellular matrix
EMT: Epithelial-mesenchymal transition
EVs: Extracellular vesicles
EV-CATCHER: Extracellular Vesicle Capture by AnTibody of CHoice and Enzymatic Release
HSC: Hepatic stellate cells
ICU: Intensive care unit
IPF: Idiopathic pulmonary fibrosis
miRNA: MicroRNA
PBMCs: Peripheral blood mononuclear cells
qPCR: Quantitative PCR
SARS-CoV-2: Severe acute respiratory syndrome coronavirus 2

## References

[1] WHO Coronavirus (COVID-19) Dashboard. 2022. https://covid19.who.int. Accessed 25 May 2022.

[2] Hasan SS, Capstick T, Ahmed R, Kow CS, Mazhar F, Merchant H (2020). Mortality in COVID-19 patients with acute respiratory distress syndrome and corticosteroids use: a systematic review and meta-analysis. Expert Rev Respir Med..

[3] Cascella M, Rajnik M, Aleem A, Dulebohn SC, Di Napoli R, Cascella M, Rajnik M, Aleem A, Dulebohn SC, Di Napoli R (2020). Features, evaluation, and treatment of Coronavirus (COVID-19). StatPearls.

[4] Grasselli G, Zangrillo A, Zanella A, Antonelli M, Cabrini L, Castelli A (2020). Baseline characteristics and outcomes of 1591 patients infected with SARS-CoV-2 admitted to ICUs of the Lombardy region, Italy. JAMA.

[5] Sanyaolu A, Okorie C, Marinkovic A, Patidar R, Younis K, Desai P (2020). Comorbidity and its Impact on Patients with COVID-19. SN Compr Clin Med.

[6] George PM, Wells AU, Jenkins RG (2020). Pulmonary fibrosis and COVID-19: the potential role for antifibrotic therapy. Lancet Respir Med.

[7] Fadista J, Kraven LM, Karjalainen J, Andrews SJ, Geller F, COVID-19 Host Genetics Initiative (2021). Shared genetic etiology between idiopathic pulmonary fibrosis and COVID-19 severity. EBioMedicine..

[8] Rai DK, Kumar S, Sahay N (2021). Post-COVID-19 pulmonary fibrosis: a case series and review of literature. J Family Med Prim Care.

[9] John AE, Joseph C, Jenkins G, Tatler AL (2021). COVID-19 and pulmonary fibrosis: a potential role for lung epithelial cells and fibroblasts. Immunol Rev.

[10] Hama Amin BJ, Kakamad FH, Ahmed GS, Ahmed SF, Abdulla BA, Mohammed SH (2022). Post COVID-19 pulmonary fibrosis; a meta-analysis study. Ann Med Surg (Lond).

[11] Alhiyari MA, Ata F, Alghizzawi MI, Bilal AB, Abdulhadi AS, Yousaf Z (2021). Post COVID-19 fibrosis, an emerging complicationof SARS-CoV-2 infection. IDCases..

[12] McGroder CF, Zhang D, Choudhury MA, Salvatore MM, D’Souza BM, Hoffman EA (2021). Pulmonary fibrosis 4 months after COVID-19 is associated with severity of illness and blood leucocyte telomere length. Thorax.

[13] Nabahati M, Ebrahimpour S, Khaleghnejad Tabari R, Mehraeen R (2021). Post-COVID-19 pulmonary fibrosis and its predictive factors: a prospective study. Egypt J Radiol Nucl Med.

[14] Aul DR, Gates DJ, Draper DA, Dunleavy DA, Ruickbie DS, Meredith DH (2021). Complications after discharge with COVID-19 infection and risk factors associated with development of post-COVID pulmonary fibrosis. Respir Med.

[15] Patil SV, Gondhali G, Patil R (2021). Post-Covid-19 lung fibrosis: study of 600 cases in tertiary care setting in India. Eur Respir J.

[16] Darcis G, Bouquegneau A, Maes N, Thys M, Henket M, Labye F (2021). Long-term clinical follow-up of patients suffering from moderate-to-severe COVID-19 infection: a monocentric prospective observational cohort study. Int J Infect Dis.

[17] Raghu G, Collard HR, Egan JJ, Martinez FJ, Behr J, Brown KK (2011). An official ATS/ERS/JRS/ALAT statement: idiopathic pulmonary fibrosis: evidence-based guidelines for diagnosis and management. Am J Respir Crit Care Med.

[18] Noble PW, Albera C, Bradford WZ, Costabel U, Glassberg MK, Kardatzke D (2011). Pirfenidone in patients with idiopathic pulmonary fibrosis (CAPACITY): two randomised trials. Lancet.

[19] King TE, Pardo A, Selman M (2011). Idiopathic pulmonary fibrosis. Lancet.

[20] Guiot J, Duysinx B, Seidel L, Henket M, Gester F, Bonhomme O (2018). Clinical experience in idiopathic pulmonary fibrosis: a retrospective study. Acta Clin Belg.

[21] Behr J, Nathan SD, Wuyts WA, Bishop NM, Bouros DE, Antoniou K (2021). Efficacy and safety of sildenafil added to pirfenidone in patients with advanced idiopathic pulmonary fibrosis and risk of pulmonary hypertension: a double-blind, randomised, placebo-controlled, phase 2b trial. Lancet Respir Med.

[22] Wynn TA (2011). Integrating mechanisms of pulmonary fibrosis. J Exp Med.

[23] Lederer DJ, Martinez FJ (2018). Idiopathic pulmonary fibrosis. N Engl J Med.

[24] Baek D, Villén J, Shin C, Camargo FD, Gygi SP, Bartel DP (2008). The impact of microRNAs on protein output. Nature.

[25] Cheng HS, Njock M-S, Khyzha N, Dang LT, Fish JE (2014). Noncoding RNAs regulate NF-κB signaling to modulate blood vessel inflammation. Front Genet.

[26] Njock M-S, Fish JE (2017). Endothelial miRNAs as cellular messengers in cardiometabolic diseases. Trends Endocrinol Metab.

[27] Njock M-S, O’Grady T, Nivelles O, Lion M, Jacques S, Cambier M (2022). Endothelial extracellular vesicles promote tumour growth by tumour-associated macrophage reprogramming. J Extracell Vesicles.

[28] Veitch S, Njock M-S, Chandy M, Siraj MA, Chi L, Mak H (2022). MiR-30 promotes fatty acid beta-oxidation and endothelial cell dysfunction and is a circulating biomarker of coronary microvascular dysfunction in pre-clinical models of diabetes. Cardiovasc Diabetol.

[29] Njock M-S, Guiot J, Henket MA, Nivelles O, Thiry M, Dequiedt F (2019). Sputum exosomes: promising biomarkers for idiopathic pulmonary fibrosis. Thorax.

[30] Parzibut G, Henket M, Moermans C, Struman I, Louis E, Malaise M (2021). A blood exosomal miRNA signature in acute respiratory distress syndrome. Front Mol Biosci.

[31] Barker KR, Lu Z, Kim H, Zheng Y, Chen J, Conroy AL (2017). miR-155 modifies inflammation, endothelial activation and blood-brain barrier dysfunction in cerebral malaria. Mol Med.

[32] Njock M-S, Cheng HS, Dang LT, Nazari-Jahantigh M, Lau AC, Boudreau E (2015). Endothelial cells suppress monocyte activation through secretion of extracellular vesicles containing antiinflammatory microRNAs. Blood.

[33] Guiot J, Cambier M, Boeckx A, Henket M, Nivelles O, Gester F (2020). Macrophage-derived exosomes attenuate fibrosis in airway epithelial cells through delivery of antifibrotic miR-142-3p. Thorax.

[34] Yang C-Y, Chen Y-H, Liu P-J, Hu W-C, Lu K-C, Tsai K-W (2022). The emerging role of miRNAs in the pathogenesis of COVID-19: protective effects of nutraceutical polyphenolic compounds against SARS-CoV-2 infection. Int J Med Sci.

[35] Marchi R, Sugita B, Centa A, Fonseca AS, Bortoletto S, Fiorentin K (2021). The role of microRNAs in modulating SARS-CoV-2 infection in human cells: a systematic review. Infect Genet Evol.

[36] Matarese A, Gambardella J, Sardu C, Santulli G (2020). miR-98 regulates TMPRSS2 Expression in human endothelial cells: key implications for COVID-19. Biomedicines..

[37] Lu D, Chatterjee S, Xiao K, Riedel I, Wang Y, Foo R (2020). MicroRNAs targeting the SARS-CoV-2 entry receptor ACE2 in cardiomyocytes. J Mol Cell Cardiol.

[38] Haddad H, Al-Zyoud W (2020). miRNA target prediction might explain the reduced transmission of SARS-CoV-2 in Jordan, Middle East. Noncoding RNA Res.

[39] Ali Hosseini Rad SM, McLellan AD (2020). Implications of sars-cov-2 mutations for genomic rna structure and host microrna targeting. Int J Mol Sci..

[40] Khan MA-A-K, Sany MRU, Islam MS, Islam AB (2020). Epigenetic regulator miRNA pattern differences among SARS-CoV, SARS-CoV-2, and SARS-CoV-2 world-wide isolates delineated the mystery behind the epic pathogenicity and distinct clinical characteristics of pandemic COVID-19. Front Genet..

[41] Chow JT-S, Salmena L (2020). Prediction and analysis of SARS-CoV-2-targeting microRNA in human lung epithelium. Genes (Basel)..

[42] Akula SM, Bolin P, Cook PP (2022). Cellular miR-150–5p may have a crucial role to play in the biology of SARS-CoV-2 infection by regulating nsp10 gene. RNA Biol..

[43] Bartoszewski R, Dabrowski M, Jakiela B, Matalon S, Harrod KS, Sanak M (2020). SARS-CoV-2 may regulate cellular responses through depletion of specific host miRNAs. Am J Physiol Lung Cell Mol Physiol.

[44] Chen L, Zhong L (2020). Genomics functional analysis and drug screening of SARS-CoV-2. Genes Dis.

[45] Ali M, Abdullah F, Naveed A, Ahmed SM, Khan AA, Hasan A (2022). Role of circulatory miRNA-21 and associated signaling pathways in the pathogenesis of pulmonary fibrosis among individuals recovered after COVID-19 infection. Human Gene.

[46] Moher D, Liberati A, Tetzlaff J, Altman DG (2010). Preferred reporting items for systematic reviews and meta-analyses: the PRISMA statement. Int J Surg.

[47] Fayyad-Kazan M, Makki R, Skafi N, El Homsi M, Hamade A, El Majzoub R (2021). Circulating miRNAs: potential diagnostic role for coronavirus disease 2019 (COVID-19). Infect Genet Evol.

[48] Kadota T, Yoshioka Y, Fujita Y, Araya J, Minagawa S, Hara H (2020). Extracellular vesicles from fibroblasts induce epithelial-cell senescence in pulmonary fibrosis. Am J Respir Cell Mol Biol.

[49] Pimenta R, Viana NI, Dos Santos GA, Candido P, Guimarães VR, Romão P (2021). MiR-200c-3p expression may be associated with worsening of the clinical course of patients with COVID-19. Mol Biol Res Commun.

[50] Mitchell MI, Ben-Dov IZ, Liu C, Ye K, Chow K, Kramer Y (2021). Extracellular Vesicle Capture by AnTibody of CHoice and Enzymatic Release (EV-CATCHER): a customizable purification assay designed for small-RNA biomarker identification and evaluation of circulating small-EVs. J Extracell Vesicles.

[51] Yang G, Yang L, Wang W, Wang J, Wang J, Xu Z (2015). Discovery and validation of extracellular/circulating microRNAs during idiopathic pulmonary fibrosis disease progression. Gene.

[52] Yamada M, Kubo H, Ota C, Takahashi T, Tando Y, Suzuki T (2013). The increase of microRNA-21 during lung fibrosis and its contribution to epithelial-mesenchymal transition in pulmonary epithelial cells. Respir Res.

[53] Yang S, Banerjee S, De Freitas A, Sanders YY, Ding Q, Matalon S (2012). Participation of miR-200 in pulmonary fibrosis. Am J Pathol.

[54] Saulle I, Garziano M, Fenizia C, Cappelletti G, Parisi F, Clerici M (2021). MiRNA profiling in plasma and placenta of SARS-CoV-2-infected pregnant women. Cells..

[55] Garg A, Seeliger B, Derda AA, Xiao K, Gietz A, Scherf K (2021). Circulating cardiovascular microRNAs in critically ill COVID-19 patients. Eur J Heart Fail.

[56] Li P, Li J, Chen T, Wang H, Chu H, Chang J (2014). Expression analysis of serum microRNAs in idiopathic pulmonary fibrosis. Int J Mol Med.

[57] Li P, Zhao G-Q, Chen T-F, Chang J-X, Wang H-Q, Chen S-S (2013). Serum miR-21 and miR-155 expression in idiopathic pulmonary fibrosis. J Asthma.

[58] Makiguchi T, Yamada M, Yoshioka Y, Sugiura H, Koarai A, Chiba S (2016). Serum extracellular vesicular miR-21-5p is a predictor of the prognosis in idiopathic pulmonary fibrosis. Respir Res.

[59] Parray A, Mir FA, Doudin A, Iskandarani A, Danjuma IMM, Kuni RAT (2021). SnoRNAs and miRNAs networks underlying COVID-19 disease severity. Vaccines (Basel).

[60] de Gonzalo-Calvo D, Benítez ID, Pinilla L, Carratalá A, Moncusí-Moix A, Gort-Paniello C (2021). Circulating microRNA profiles predict the severity of COVID-19 in hospitalized patients. Transl Res.

[61] Lino Cardenas CL, Henaoui IS, Courcot E, Roderburg C, Cauffiez C, Aubert S (2013). miR-199a-5p Is upregulated during fibrogenic response to tissue injury and mediates TGFbeta-induced lung fibroblast activation by targeting caveolin-1. PLoS Genet.

[62] Demiray A, Sarı T, Çalışkan A, Nar R, Aksoy L, Akbubak İH (2021). Serum microRNA signature is capable of predictive and prognostic factor for SARS-COV-2 virulence. Turk J Biochem.

[63] Mullenbrock S, Liu F, Szak S, Hronowski X, Gao B, Juhasz P (2018). Systems analysis of transcriptomic and proteomic profiles identifies novel regulation of fibrotic programs by miRNAs in pulmonary fibrosis fibroblasts. Genes (Basel).

[64] Li C-X, Chen J, Lv S-K, Li J-H, Li L-L, Hu X (2021). Whole-transcriptome RNA sequencing reveals significant differentially expressed mRNAs, miRNAs, and lncRNAs and related regulating biological pathways in the peripheral blood of COVID-19 patients. Mediators Inflamm.

[65] Berschneider B, Ellwanger DC, Baarsma HA, Thiel C, Shimbori C, White ES (2014). miR-92a regulates TGF-β1-induced WISP1 expression in pulmonary fibrosis. Int J Biochem Cell Biol.

[66] Gregory PA, Bert AG, Paterson EL, Barry SC, Tsykin A, Farshid G (2008). The miR-200 family and miR-205 regulate epithelial to mesenchymal transition by targeting ZEB1 and SIP1. Nat Cell Biol.

[67] Chen Z, Wang X, Li L, Han M, Wang M, Li Z (2021). Construction of an autophagy interaction network based on competitive endogenous RNA reveals the key pathways and central genes of SARS-CoV-2 infection in vivo. Microb Pathog.

[68] Qian W, Cai X, Qian Q, Peng W, Yu J, Zhang X (2019). lncRNA ZEB1-AS1 promotes pulmonary fibrosis through ZEB1-mediated epithelial–mesenchymal transition by competitively binding miR-141-3p. Cell Death Dis.

[69] Huang C, Xiao X, Yang Y, Mishra A, Liang Y, Zeng X (2017). MicroRNA-101 attenuates pulmonary fibrosis by inhibiting fibroblast proliferation and activation. J Biol Chem.

[70] Lacedonia D, Scioscia G, Soccio P, Conese M, Catucci L, Palladino GP (2021). Downregulation of exosomal let-7d and miR-16 in idiopathic pulmonary fibrosis. BMC Pulm Med.

[71] Ji X, Wu B, Fan J, Han R, Luo C, Wang T (2015). The anti-fibrotic effects and mechanisms of microRNA-486-5p in pulmonary fibrosis. Sci Rep.

[72] Liu B, Li R, Zhang J, Meng C, Zhang J, Song X (2018). MicroRNA-708-3p as a potential therapeutic target via the ADAM17-GATA/STAT3 axis in idiopathic pulmonary fibrosis. Exp Mol Med.

[73] Casanova NG, Zhou T, Gonzalez-Garay ML, Lussier YA, Sweiss N, Ma S-F (2021). MicroRNA and protein-coding gene expression analysis in idiopathic pulmonary fibrosis yields novel biomarker signatures associated to survival. Transl Res.

[74] Chen Y, Zhao X, Sun J, Su W, Zhang L, Li Y (2019). YAP1/Twist promotes fibroblast activation and lung fibrosis that conferred by miR-15a loss in IPF. Cell Death Differ.

[75] Farr RJ, Rootes CL, Rowntree LC, Nguyen THO, Hensen L, Kedzierski L (2021). Altered microRNA expression in COVID-19 patients enables identification of SARS-CoV-2 infection. PLoS Pathog.

[76] Xie T, Liang J, Geng Y, Liu N, Kurkciyan A, Kulur V (2017). MicroRNA-29c prevents pulmonary fibrosis by regulating epithelial cell renewal and apoptosis. Am J Respir Cell Mol Biol.

[77] Tang H, Gao Y, Li Z, Miao Y, Huang Z, Liu X (2020). The noncoding and coding transcriptional landscape of the peripheral immune response in patients with COVID-19. Clin Transl Med.

[78] Nho RS, Im J, Ho Y-Y, Hergert P (2014). MicroRNA-96 inhibits FoxO3a function in IPF fibroblasts on type I collagen matrix. Am J Physiol Lung Cell Mol Physiol.

[79] Disayabutr S, Kim EK, Cha S-I, Green G, Naikawadi RP, Jones KD (2016). miR-34 miRNAs regulate cellular senescence in type II alveolar epithelial cells of patients with idiopathic pulmonary fibrosis. PLoS ONE.

[80] Donyavi T, Bokharaei-Salim F, Baghi HB, Khanaliha K, Alaei Janat-Makan M, Karimi B (2021). Acute and post-acute phase of COVID-19: Analyzing expression patterns of miRNA-29a-3p, 146a–3p, 155–5p, and let-7b-3p in PBMC. Int Immunopharmacol.

[81] Grehl C, Schultheiß C, Hoffmann K, Binder M, Altmann T, Grosse I (2021). Detection of SARS-CoV-2 derived small RNAs and changes in circulating small RNAs associated with COVID-19. Viruses..

[82] Keikha R, Hashemi-Shahri SM, Jebali A (2021). The relative expression of miR-31, miR-29, miR-126, and miR-17 and their mRNA targets in the serum of COVID-19 patients with different grades during hospitalization. Eur J Med Res.

[83] Tsitoura E, Wells AU, Karagiannis K, Lasithiotaki I, Vasarmidi E, Bibaki E (2016). MiR-185/AKT and miR-29a/collagen 1a pathways are activated in IPF BAL cells. Oncotarget.

[84] Bagnato G, Roberts WN, Roman J, Gangemi S (2017). A systematic review of overlapping microRNA patterns in systemic sclerosis and idiopathic pulmonary fibrosis. Eur Respir Rev.

[85] Korpal M, Lee ES, Hu G, Kang Y (2008). The miR-200 family inhibits epithelial-mesenchymal transition and cancer cell migration by direct targeting of E-cadherin transcriptional repressors ZEB1 and ZEB2. J Biol Chem.

[86] Liu G, Friggeri A, Yang Y, Milosevic J, Ding Q, Thannickal VJ (2010). miR-21 mediates fibrogenic activation of pulmonary fibroblasts and lung fibrosis. J Exp Med.

[87] Zhou J, Xu Q, Zhang Q, Wang Z, Guan S (2018). A novel molecular mechanism of microRNA-21 inducing pulmonary fibrosis and human pulmonary fibroblast extracellular matrix through transforming growth factor β1-mediated SMADs activation. J Cell Biochem.

[88] Yang S, Cui H, Xie N, Icyuz M, Banerjee S, Antony VB (2013). MiR-145 regulates myofibroblast differentiation and lung fibrosis. FASEB J.

[89] Wu J, Huang Q, Li P, Wang Y, Zheng C, Lei X (2019). MicroRNA-145 promotes the epithelial-mesenchymal transition in peritoneal dialysis-associated fibrosis by suppressing fibroblast growth factor 10. J Biol Chem.

[90] Messner CJ, Schmidt S, Özkul D, Gaiser C, Terracciano L, Krähenbühl S (2021). Identification of miR-199a-5p, miR-214-3p and miR-99b-5p as fibrosis-specific extracellular biomarkers and promoters of HSC activation. Int J Mol Sci.

[91] Xiao X, Huang C, Zhao C, Gou X, Senavirathna LK, Hinsdale M (2015). Regulation of myofibroblast differentiation by miR-424 during epithelial-to-mesenchymal transition. Arch Biochem Biophys.

[92] Huang Y, Xie Y, Abel PW, Wei P, Plowman J, Toews ML (2020). TGF-β1-induced miR-424 promotes pulmonary myofibroblast differentiation by targeting Slit2 protein expression. Biochem Pharmacol.

[93] He L, Thomson JM, Hemann MT, Hernando-Monge E, Mu D, Goodson S (2005). A microRNA polycistron as a potential human oncogene. Nature.

[94] Ota A, Tagawa H, Karnan S, Tsuzuki S, Karpas A, Kira S (2004). Identification and characterization of a novel gene, C13orf25, as a target for 13q31-q32 amplification in malignant lymphoma. Cancer Res.

[95] Dakhlallah D, Batte K, Wang Y, Cantemir-Stone CZ, Yan P, Nuovo G (2013). Epigenetic regulation of miR-17~92 contributes to the pathogenesis of pulmonary fibrosis. Am J Respir Crit Care Med.

[96] Xu T, Jing C, Shi Y, Miao R, Peng L, Kong S (2015). microRNA-20a enhances the epithelial-to-mesenchymal transition of colorectal cancer cells by modulating matrix metalloproteinases. Exp Ther Med.

[97] Sing T, Jinnin M, Yamane K, Honda N, Makino K, Kajihara I (2012). microRNA-92a expression in the sera and dermal fibroblasts increases in patients with scleroderma. Rheumatology (Oxford).

[98] Liu P, Su J, Song X, Wang S (2018). miR-92a regulates the expression levels of matrix metalloproteinase 9 and tissue inhibitor of metalloproteinase 3 via sirtuin 1 signaling in hydrogen peroxide-induced vascular smooth muscle cells. Mol Med Rep.

[99] Huang Y, Tong J, He F, Yu X, Fan L, Hu J (2015). miR-141 regulates TGF-β1-induced epithelial-mesenchymal transition through repression of HIPK2 expression in renal tubular epithelial cells. Int J Mol Med.

[100] Wang H, Zhang Y, Wu Q, Wang Y-B, Wang W (2018). miR-16 mimics inhibit TGF-β1-induced epithelial-to-mesenchymal transition via activation of autophagy in non-small cell lung carcinoma cells. Oncol Rep.

[101] Inomata M, Kamio K, Azuma A, Matsuda K, Usuki J, Morinaga A (2021). Rictor-targeting exosomal microRNA-16 ameliorates lung fibrosis by inhibiting the mTORC2-SPARC axis. Exp Cell Res.

[102] Guo C-J, Pan Q, Li D-G, Sun H, Liu B-W (2009). miR-15b and miR-16 are implicated in activation of the rat hepatic stellate cell: an essential role for apoptosis. J Hepatol.

[103] Pan Q, Guo C-J, Xu Q-Y, Wang J-Z, Li H, Fang C-H (2020). miR-16 integrates signal pathways in myofibroblasts: determinant of cell fate necessary for fibrosis resolution. Cell Death Dis.

[104] Yao Q, Xing Y, Wang Z, Liang J, Lin Q, Huang M (2020). MiR-16-5p suppresses myofibroblast activation in systemic sclerosis by inhibiting NOTCH signaling. Aging (Albany NY).

[105] Bo Y, Liu B, Yang L, Zhang L, Yan Y (2021). Exosomes derived from miR-16-5p-overexpressing keratinocytes attenuates bleomycin-induced skin fibrosis. Biochem Biophys Res Commun.

[106] Su S, Zhao Q, He C, Huang D, Liu J, Chen F (2015). miR-142-5p and miR-130a-3p are regulated by IL-4 and IL-13 and control profibrogenic macrophage program. Nat Commun.

[107] Morizane R, Fujii S, Monkawa T, Hiratsuka K, Yamaguchi S, Homma K (2014). miR-34c attenuates epithelial-mesenchymal transition and kidney fibrosis with ureteral obstruction. Sci Rep.

[108] Wang Y, Ouyang M, Wang Q, Jian Z (2016). MicroRNA-142-3p inhibits hypoxia/reoxygenation-induced apoptosis and fibrosis of cardiomyocytes by targeting high mobility group box 1. Int J Mol Med.

[109] Yang X, Dan X, Men R, Ma L, Wen M, Peng Y (2017). MiR-142-3p blocks TGF-β-induced activation of hepatic stellate cells through targeting TGFβRI. Life Sci.

[110] Sato S, Chong SG, Upagupta C, Yanagihara T, Saito T, Shimbori C (2021). Fibrotic extracellular matrix induces release of extracellular vesicles with pro-fibrotic miRNA from fibrocytes. Thorax.

[111] Cui H, Banerjee S, Xie N, Ge J, Liu R-M, Matalon S (2016). MicroRNA-27a-3p is a negative regulator of lung fibrosis by targeting myofibroblast differentiation. Am J Respir Cell Mol Biol.

