# Supplementary material for: Systematic review of overlapping microRNA patterns in COVID-19 and idiopathic pulmonary fibrosis
Source: Respir Res. 2023 Apr 15;24:112. doi: 10.1186/s12931-023-02413-6 (PMC10105547; doi:10.1186/s12931-023-02413-6)
Supplement: Supplementary file 2 — Additional file 2: Table S1. miRNA patterns in COVID-19 [file 12931_2023_2413_MOESM2_ESM.docx]

**Additional file 2: Table S1: miRNA patterns in COVID-19**

| Study | miRNAs (regulation) | Biological material | Sujects (n) | Methods | Outcome summary |
| --- | --- | --- | --- | --- | --- |
| JT McDonald *et al.* (2021) [1] | miR-2392 (↑)  miR-1-3p (↓) and  miR-155-5p (↓) | Serum, urine  Serum | COVID-19=10, healthy controls=10 | ddPCR, miR-2392 mimic, anti miR-2392 inhibitor | miR-2392 induces the suppression of mitochondrial gene expression, which would have the most adverse effects on the high mitochondrial energetic tissues (brain, heart, and kidney); increase of inflammation, glycolysis, and hypoxia |
| R Pimenta et al. (2021) [2] | miR-200c-3p (↑) | Saliva | COVID-19=72, controls=39 | qPCR | miR-200c-3p is a predictor of severity independent of COVID-19 risk factors |
| C Grehl *et al.* (2021) [3] | miR-4516 (↑), miR-362-5p (↑), miR-548 (↑), miR-320a-3p (↑), miR-320b (↑), miR-320c (↑), miR-320d (↑), miR-185-5p (↑), miR-629-5p (↑),  miR-122-3p (↓), miR-146b-5p (↓), miR-29a-3p (↓), miR-342-3p (↓) | Plasma | COVID-19 patients:  severe (n=5) *vs*  mild (n=3) | small-RNA Sequencing | Several of these miRNAs are associated with JAK-STAT response and cytokine storm |
| I Saulle et al. (2021) [4] | miR-21 (↑), miR-23b (↑), miR-28 (↑), miR-29a (↑), miR-29c (↑), miR-98 (↑), miR-326 (↑), miR-17 (↑), miR-92 (↑), miR-146 (↑), miR-150 (↑), miR-155 (↑), miR-223 (↑)  miR-21b (↑), miR-29c (↑), miR-98 (↑), miR-146 (↑),miR-150 (↑), miR-155 (↑), miR-190 (↑), miR-346 (↑), miR-326 (↑) | Plasma  Placenta | COVID-19=15, controls=6 | qPCR array | Combination of dysregulated miRNAs and antiviral/immune factors seems to control both the infection and the dysfunctional immune reaction |
| M Fayyad-Kazan *et al.* (2021) [5] | miR-15a-5p (↑), miR-19a-3p (↑), miR-19b-3p (↑), miR-23a-3p (↑), miR-92a-3p (↑), miR-320a (↑), miR-17-5p (↓), miR-142-5p (↓) | Plasma | COVID-19=6, healthy controls=6 | qPCR array, qPCR | Plasma miR-19a-3p, miR-19b-3p, and miR-92a-3p expression levels could serve as potential diagnostic biomarker for SARS-CoV-2-infection |
| R Keikha et al. (2021) [6] | miR-17-3p (↑)  miR-31-3p (↓), hsa-miR-29a-3p (↓), and miR-126-3p (↓) | Serum | COVID-19 patients with grade 1 (n=21), grade 2 (n=20), grade 3 (n =20), grade 4 (n=21), and grade 5 (n=21) | qPCR | Relative expression of miR-31-3p, miR-29a-3p, and miR-126-3p was down-regulated and relative expression of miR-17-3p was up-regulated with the increase of COVID-19 grade |
| Z Chen et al. (2021) [7] | miR-1226–3p (↑), miR-103a-2-5p (↑), miR-485–5p (↑), miR-652–3p (↑), let-7d-3p (↑), miR-3614–5p (↑), miR-3194–3p (↓), miR-514a-3p (↓), miR-1291 (↓), miR-142-3p (↓), miR-340–3p (↓), miR-708–3p (↓), miR-511–5p (↓), miR-141–3p (↓), miR-548ar-3p (↓), miR-34c-5p (↓), miR-184 (↓), miR-4772–5p (↓), miR-27a-3p (↓), miR-642a-5p (↓), miR-192–5p (↓) | PBMCs | COVID-19=17, healthy controls=6 | small-RNA Sequencing | miR-340–3p, miR-652–3p, miR-4772–5p, miR-192–5p may be biomarkers that predict changes in mild SARS-CoV-2 infection. Some molecules, including hsa-miR-1291, were considered potential targets to predict the emergence of severe symptoms in SARS-CoV-2 infection |
| MI Mitchell et al. (2021) [8] | miR-15a (↑), miR-424 (↑), miR-627-5p (↑), miR-145 (↑), miR-205 (↑), and miR-200c (↑), miR-146a (↓), miR-126-3p (↓), miR-151-3p (↓), miR-126-5p (↓)  miR-550-5p (↓), miR-629* (↓) | Serum-derived EVs  Whole serum | COVID-19 patients:  severe (n=17) *vs* mild (n=13) | EV-CATCHER, small-RNA Sequencing, qPCR | miR-146a and miR-126-3p are significantly downregulated in serum-derived EVs with disease severity |
| M Safdar et al. (2021) [9] | miR‑6741‑3p (↑) | Saliva | COVID-19=20, healthy controls=20 | qPCR | An effective association between miR-6741-3p and kidney diseases, and their associated SARS-COV-2 infection |
| D de Gonzalo-Calvo et al. (2021) [10] | miR-27a-3p (↑), miR-27b-3p (↑), miR-148a-3p (↑), miR-199a-5p (↑), miR-491-5p (↑), miR-16-5p (↓), miR-92a-3p (↓), miR-150-5p (↓), miR-451a (↓), miR-486-5p (↓) | Plasma | COVID-19 patients: ICU (n=36) *vs* ward (n=43) | qPCR array | Signature of three miRNAs (miR-148a-3p, miR-451a and miR-486-5p) that distinguishes between ICU and ward patients |
| T Donyavi et al. (2021) [11] | miR-29a-3p (↑), miR-146a-3p (↑), miR-155-5p (↑), let-7b-3p (↑) | PBMCs | COVID-19=18, healthy controls=15 | qPCR | miR-29a-3p, miR-155-5p and miR-146a-3p may serve as the novel biomarker for COVID-19 diagnosis |
| Z Bagheri-Hosseinabadi et al. (2021) [12] | miR-10b (↓) | Serum | COVID-19=33, healthy controls=29 | qPCR | miR-10b is downregulated in the COVID-19 patients and might result in increased levels of IL-2 and IL-8, hence contributing to cytokine storm |
| S Li et al. (2021) [13] | miR-155 (↑), miR-130a (↑) | Blood | COVID-19=30, healthy controls=24 | qPCR | miR-155 and miR-130a levels were markedly higher in COVID-19 patients with mild/moderate compared to the severe/critical group. |
| J Sabbatinelli et al. (2021) [14] | miR-146a-5p (↓) | Serum | COVID-19=29, healthy controls=29 | ddPCR, qPCR | miR-146a-5p can provide clues about the molecular link between inflammaging and COVID-19 clinical course |
| H Tang et al. (2020) [15] | miR-3605-3p (↑), miR-15b-5p (↑), miR-486-5p (↑), miR-486-3p (↑), miR-99a-5p (↑), miR-146a-5p (↓), miR-21-5p (↓), miR-142-3p (↓), miR-181a-2-3p (↓), miR31-5p (↓) | Whole blood | COVID-19 patients:  severe (n=6) *vs*  moderate (n=6) | small-RNA Sequencing | miR-146a-5p, miR-21-5p, miR-142-3p, and miR-15b-5p are potential contributors to the disease pathogenesis, possibly serving as biomarkers of severe COVID-19. |
| RP Duecker et al. (2021) [16] | miR-320a (↓), miR-320b (↓), miR-320c (↓) | Blood | COVID-19=21 (severe, n=10; moderate, n=11), healthy controls=8 | small-RNA Sequencing | miRNA-320 family members (miR-320a, miR-320b and miR-320c) as potential biomarkers for severe progressive SARS-CoV-2 infection |
| A Parray et al. (2021) [17] | miR-1246 (↑), miR-4532 (↑), miR-145-5p (↑), miR-3651 (↑), let-7i-5p (↓), miR-3180-3p (↓)  miR-145-5p (↑), miR-3651 (↑),miR-3609 (↑), let-7i-5p (↓), miR-1231 (↓),miR-885-3p (↓) | Blood | COVID-19 patients:  severe (n=9) vs  asymptomatic(n=10)  severe (n=9) vs  mild (n=10) | microarray | Unique miRNA and snoRNA profile that is associated with a higher risk of severity in a cohort of SARS-CoV-2 infected patients |
| RJ Farr et al. (2021) [18] | miR-4742-3p (↑), miR-31-5p (↑), miR-3215-3p (↑), miR-423-5p (↑), miR-195-5p (↑), miR-776-3p (↓) and miR-1275 (↓) | Plasma | COVID-19=10, healthy controls=10 | small-RNA Sequencing, qPCR | miRNA signature, consisting of miR423-5p, miR-23a-3p, miR-195-5p, could independently classify COVID-19 patients from healthy controls (99.9% accuracy) |
| A Demiray et al. (2021) [19] | miR-190a (↑), miR-203 (↑), let-7d (↓), miR-17 (↓), miR-34b (↓), miR-93 (↓), miR-200b (↓), miR-200c (↓), miR-223 (↓) | Serum | COVID-19=40, healthy controls=10 | qPCR | The increase in miR-190a level may be a prognostic factor related to the COVID-19 disease |
| A Garg et al. (2021) [20] | miR-21 (↑), miR-155 (↑), miR-208a (↑) and miR-499 (↑) | Serum | COVID-19=38, healthy controls=47 | qPCR | Profiles of inflammation (miR-155)- and cardiac myocyte-associated miRNAs (miR-499, miR208a) were able to differentiate between severely ill, mechanically-ventilated influenza-ARDS and COVID-19 patients, indicating a rather specific response and cardiac involvement of COVID-19. |
| CX Li et al. (2021) [21] | miR-16-2-3p (↑), miR-5695 (↑), miR-10399-3p (↑), miR-6501-5p (↑) miR-4659a-3p (↑), miR-142-5p (↑), miR-505-5p (↑), miR-125b-5p (↑), miR-618 (↑), miR-183-5p (↓), miR-627-5p (↓), miR-21-5p (↓), miR-20a-5p(↓), miR-18a-5p (↓), miR-4521 (↓), miR-144-3p (↓), miR-199a-3p (↓), miR-199b-3p (↓), miR-96-5p (↓) | Blood | COVID-19=10, healthy controls=4 | small-RNA Sequencing | New insights into inflammation regulatory mechanisms of miRs in COVID-19, which may provide novel diagnostic biomarkers and therapeutic avenues for COVID-19 patients |

***Abbreviations: COVID-19****: Coronavirus disease 2019;* ***ddPCR****: Droplet digital PCR;* ***EVs****: Extracellular vesicles ;* ***EV-CATCHER****: Extracellular Vesicle Capture by AnTibody of CHoice and Enzymatic Release;* ***ICU****: Intensive care unit;* ***PBMCs****: Peripheral blood mononuclear cells;* ***qPCR****: quantitative PCR;* ***SARS-CoV-2****: severe acute respiratory syndrome coronavirus 2;* ***↑****: high levels;* ***↓****: low levels.*

**REFERENCES:**

1. McDonald JT, Enguita FJ, Taylor D, Griffin RJ, Priebe W, Emmett MR, et al. Role of miR-2392 in driving SARS-CoV-2 infection. Cell Rep. 2021;109839.

2. Pimenta R, Viana NI, Dos Santos GA, Candido P, Guimarães VR, Romão P, et al. MiR-200c-3p expression may be associated with worsening of the clinical course of patients with COVID-19. Mol Biol Res Commun. 2021;10:141–7.

3. Grehl C, Schultheiß C, Hoffmann K, Binder M, Altmann T, Grosse I, et al. Detection of SARS-CoV-2 Derived Small RNAs and Changes in Circulating Small RNAs Associated with COVID-19. Viruses. 2021;13.

4. Saulle I, Garziano M, Fenizia C, Cappelletti G, Parisi F, Clerici M, et al. MiRNA Profiling in Plasma and Placenta of SARS-CoV-2-Infected Pregnant Women. Cells. 2021;10.

5. Fayyad-Kazan M, Makki R, Skafi N, El Homsi M, Hamade A, El Majzoub R, et al. Circulating miRNAs: Potential diagnostic role for coronavirus disease 2019 (COVID-19). Infect Genet Evol. 2021;94:105020.

6. Keikha R, Hashemi-Shahri SM, Jebali A. The relative expression of miR-31, miR-29, miR-126, and miR-17 and their mRNA targets in the serum of COVID-19 patients with different grades during hospitalization. Eur J Med Res. 2021;26:75.

7. Chen Z, Wang X, Li L, Han M, Wang M, Li Z, et al. Construction of an autophagy interaction network based on competitive endogenous RNA reveals the key pathways and central genes of SARS-CoV-2 infection in vivo. Microb Pathog. 2021;158:105051.

8. Mitchell MI, Ben-Dov IZ, Liu C, Ye K, Chow K, Kramer Y, et al. Extracellular Vesicle Capture by AnTibody of CHoice and Enzymatic Release (EV-CATCHER): A customizable purification assay designed for small-RNA biomarker identification and evaluation of circulating small-EVs. J Extracell Vesicles. 2021;10:e12110.

9. Safdar M, Khan MS, Karim AY, Omar SA, Smail SW, Saeed M, et al. SNPs at 3’UTR of APOL1 and miR-6741-3p target sites associated with kidney diseases more susceptible to SARS-COV-2 infection: in silco and in vitro studies. Mamm Genome. 2021;32:389–400.

10. Gonzalo-Calvo D de, Benítez ID, Pinilla L, Carratalá A, Moncusí-Moix A, Gort-Paniello C, et al. Circulating microRNA profiles predict the severity of COVID-19 in hospitalized patients. Transl Res. 2021;236:147–59.

11. Donyavi T, Bokharaei-Salim F, Baghi HB, Khanaliha K, Alaei Janat-Makan M, Karimi B, et al. Acute and post-acute phase of COVID-19: Analyzing expression patterns of miRNA-29a-3p, 146a-3p, 155-5p, and let-7b-3p in PBMC. Int Immunopharmacol. 2021;97:107641.

12. Bagheri-Hosseinabadi Z, Ostad Ebrahimi H, Bahrehmand F, Taghipour G, Abbasifard M. The relationship between serum levels of interleukin-2 and IL-8 with circulating microRNA-10b in patients with COVID-19. Iran J Immunol. Iran; 2021;18:65–73.

13. Li S, Duan X, Li Y, Li M, Gao Y, Li T, et al. Differentially expressed immune response genes in COVID-19 patients based on disease severity. Aging (Albany NY). 2021;13:9265–76.

14. Sabbatinelli J, Giuliani A, Matacchione G, Latini S, Laprovitera N, Pomponio G, et al. Decreased serum levels of the inflammaging marker miR-146a are associated with clinical non-response to tocilizumab in COVID-19 patients. Mech Ageing Dev. 2021;193:111413.

15. Tang H, Gao Y, Li Z, Miao Y, Huang Z, Liu X, et al. The noncoding and coding transcriptional landscape of the peripheral immune response in patients with COVID-19. Clin Transl Med. 2020;10:e200.

16. Duecker RP, Adam EH, Wirtz S, Gronau L, Khodamoradi Y, Eberhardt FJ, et al. The MiR-320 Family Is Strongly Downregulated in Patients with COVID-19 Induced Severe Respiratory Failure. Int J Mol Sci. 2021;22:10351.

17. Parray A, Mir FA, Doudin A, Iskandarani A, Danjuma IMM, Kuni RAT, et al. SnoRNAs and miRNAs Networks Underlying COVID-19 Disease Severity. Vaccines (Basel). 2021;9:1056.

18. Farr RJ, Rootes CL, Rowntree LC, Nguyen THO, Hensen L, Kedzierski L, et al. Altered microRNA expression in COVID-19 patients enables identification of SARS-CoV-2 infection. PLoS Pathog. 2021;17:e1009759.

19. Demiray A, Sarı T, Çalışkan A, Nar R, Aksoy L, Akbubak İH. Serum microRNA signature is capable of predictive and prognostic factor for SARS-COV-2 virulence. Turk J Biochem. 2021;46:245.

20. Garg A, Seeliger B, Derda AA, Xiao K, Gietz A, Scherf K, et al. Circulating cardiovascular microRNAs in critically ill COVID-19 patients. Eur J Heart Fail. 2021;23:468–75.

21. Li C-X, Chen J, Lv S-K, Li J-H, Li L-L, Hu X. Whole-Transcriptome RNA Sequencing Reveals Significant Differentially Expressed mRNAs, miRNAs, and lncRNAs and Related Regulating Biological Pathways in the Peripheral Blood of COVID-19 Patients. Mediators Inflamm. 2021;2021:6635925.
