# Supplementary material for: Systematic review of overlapping microRNA patterns in COVID-19 and idiopathic pulmonary fibrosis
Source: Respir Res. 2023 Apr 15;24:112. doi: 10.1186/s12931-023-02413-6 (PMC10105547; doi:10.1186/s12931-023-02413-6)
Supplement: Supplementary file 3 — Additional file 3: Table S2. miRNA patterns in IPF [file 12931_2023_2413_MOESM3_ESM.docx]

**Additional file 3: Table S2: miRNA patterns in IPF**

| Study | miRNAs (regulation) | Biological material | Sujects (n) | Methods | Outcome summary |
| --- | --- | --- | --- | --- | --- |
| D Lacedonia et al. (2021) [1] | Let-7d (↓), miR-16 (↓) | Serum-derived exosomes | IPF=61, healthy controls=15 | qPCR | Identification of new key players (Let-7d, miR-16) in the pathophysiology of IPF. |
| CM Prêle et al. (2021) [2] | miR-155 (↑)  miR-155 (↓) | IPF tissue  IPF fibroblasts | IPF=6, healthy controls=9 | qPCR | Although miR-155 levels were increased in IPF tissue, they were reduced in IPF fibroblasts. |
| S Sato et al. [3] | miR-21 (↑) | IPF fibrocytes (BALF), EVs |  | qPCR | Fibrocytes from BALF collected from fibrotic interstitial pneumonia patients showed higher miR-21-5 p expression than those from other patients. |
| GE Shochet (2021) [4] | miR-608 (↑) | Lung tissue (FFPE) | IPF=18, controls=8 | qPCR | miR-608 is overexpressed in IPF patients. |
| NG Casanova et al. (2021) [5] | miR-223 (↑), miR-1275 (↑), miR-125b-1* (↑), miR-144 (↑), miR-31 (↓), miR-342-3p (↓), miR-150 (↓), miR-342-5p (↓), miR-548e (↓), miR-636 (↓), miR-891a (↓), miR-196a* (↓) | PBMCs | IPF=70 (according to disease severity) | MiRNA qPCR array | miRNA-driven peripheral blood molecular signatures as valuable and novel biomarkers associated to individuals at high survival risk and for potentially facilitating individualized therapies in IPF disease. |
| J Guiot et al. (2020) [6] | miR-142-3p (↑), miR-33a-5p (↑), Let-7d-5p (↓)  miR-142-3p (↑), miR-200c-5p (↑) | Sputum-derived exosomes  Plasma-derived exosomes | IPF=19, healthy controls=23  IPF=14, healthy controls=14 | qPCR, miR-142-3p mimic, miR-142-3p inhibitor | Macrophage-derived exosomes may fight against pulmonary fibrosis progression via the delivery of antifibrotic miR-142–3 p to alveolar epithelial cells and lung fibroblasts |
| Q Li et al. (2020) [7] | miR‑124 (↑), miR‑524‑5p (↑), miR‑194 (↓), miR‑133a (↓) | Lung tissue (FFPE) | IPF=20, healthy controls=8 | qPCR | miR-124, miR-524-5p, miR-194 and miR-133a were differentially expressed between patients with IPF and age-matched men without fibrotic lung disease |
| T Kadota et al. (2020) [8] | miR-19a (↑), miR-23b (↑), miR-127 (↑), miR-145 (↑), miR-424 (↑), miR-494 (↑) | Lung fibroblast-derived EVs | IPF=20, healthy controls=26 | Microarray, qPCR, miRNA inhibitor, siRNAs | IPF lung fibroblast-derived EVs contain elevated levels of miR-145, miR-23b-3p and miR-494-3p, inducing epithelial - cell senescence by targeting SIRT3, indeed acting as paracrine mediator in the pathogenesis of IPF. |
| Y Chen et al. (2019) [9] | miR-15a (↓), miR-15b (↓), miR-497 (↓) | Lung tissue | IPF=106, healthy controls=50 | Microarray, GEO, miR-15a mimic, miR-15a inhibitor | miR-15a inhibits fibrogenesis in lung fibroblast and abrogated BLM-induced lung fibrosis in mice. Novel strategies for the prevention and treatment of lung fibrosis. |
| M-S Njock et al. (2019) [10] | miR-142-3p (↑), miR-33a-5p (↑), miR-192–5p (↑), let-7d-5p (↓), miR-26a-5p (↓), miR-29b-3p (↓), miR-423-3p (↓) | Sputum-derived exosomes | IPF=16, healthy controls=14 | MiRNA qPCR array | First characterisation of miRNA content of sputum-derived exosomes in IPF that identified promising biomarkers for diagnosis and disease severity. |
| S Mullenbrock et al. (2018) [11] | miR-93-5p (↑), miR-335-5p (↑), -miR-30a-3p (↑), miR-574-3p (↑), miR-138-5p (↓), miR-146b-5p (↓), miR-155-5p (↓), miR-190a-5p (↓), miR-125b-2-3p (↓), miR-708-3p (↓), miR-20a-5p (↓), miR-374a-3p (↓), miR-21-3p (↓), miR-340-5p (↓), miR-17-5p (↓), miR-1307-5p (↓) | Lung fibroblasts | IPF=10, healthy controls=10 | miRNA-seq, miR-29b-3p, miR-146b-5p, or miR-138-5p mimics | Over expression of miR-29b-3p, miR-146b-5p, or miR-138-5p decreased expression of distinct sets of fibrotic signature genes. |
| B Liu et al. (2018) [12] | miR‑125b (↑), miR‑128 (↑), miR‑21 (↑), miR‑100 (↑), miR‑140‑3p (↑), miR‑374b (↑), let‑7d (↓), miR‑103 (↓), miR‑26 (↓), miR-30a-5p (↓) | BALF | IPF=30, healthy controls=16 | Microarray, miR-30a-5p mimic | Decreased expression of miR‑30a in the BALF of patients with IPF, along with the consequential increase in TAB3 expression, may be a crucial factor in IPF progression. |
| B Liu et al. (2018) [13] | miR-708-3p (↓) | PBMCs | IPF=78, healthy controls=78 | qPCR | Downregulation of miR-708-3p aggravates IPF, and miR-708-3p can serve as a potential therapeutic target for IPF. |
| R Li et al (2018) [14] | miR‑630 (↑), miR‑324‑5p (↑) | Plasma | IPF=10, healthy controls=10 | qPCR |  |
| T Xie et al. (2017) [15] | miR-29c (↓) | Alveolar epithelial cells (AECs) | IPF=7, healthy controls=4 | qPCR, miR-29c-3p overexpression | miR-29c maintains epithelial integrity and promotes recovery from lung injury, thereby attenuating lung fibrosis in mice. |
| C Huang et al. (2017) [16] | miR-124 (↑), miR-493a (↑), miR-1249 (↑), miR-101 (↓), miR-142-5p (↓), miR-141 (↓), miR-519d (↓), miR-877 (↓), miR-597 (↓), miR-887 (↓), miR-888a (↓), miR-1274b (↓), miR-195 (↓), miR-27b (↓) | Lung | IPF=28 (<50% FVC *vs*  >80% FVC) | Microarray, qPCR | miR-101 is an antifibrotic microRNA and a potential therapeutic target for pulmonary fibrosis. |
| H Cui et al. (2017) [17] | miR-34a (↑) | Lung | IPF=14, healthy controls=10 | qPCR, miR-34a inhibition | miR-34a functions through a negative feedback mechanism to restrain fibrotic response in the lungs by promoting senescence of pulmonary fibroblasts |
| SK Shetty et al. (2017) [18] | miR-34a (↑) | Lung (AECs) | IPF=4, healthy controls=4 | qPCR, miR-34a inhibition and overexpression | p53-miR-34a feedback as a potential therapeutic target in pulmonary fibrosis. |
| S Zhang et al. (2017) [19] | miR-30a (↓) | Plasma | IPF=46, healthy controls=46 | qPCR, miR-30a mimic and inhibitor | miR-30a could act as a potential therapeutic target for IPF. |
| E Tsitoura et al. (2016) [20] | miR-29a (↓), miR-185 (↓) | BAL cells | IPF=45, healthy controls=17 | qPCR, miR- mimic and inhibitor | Novel evidence of the involvement of the miR-185/AKT pathway in IPF BAL cells, and support for the use of miR-29a and miR-185 as BAL IPF biomarkers |
| T Makiguchi et al. (2016) [21] | miR-21-5p (↑) | Serum-derived EVs | IPF=41, healthy controls=21 | qPCR | EV miR-21-5p as potential prognostic biomarker for IPF. |
| GS Lei et al. (2016) [22] | miR-185 (↓), miR-186 (↓) | Lung | IPF=15, healthy controls=15 | qPCR, miR-185 and miR-186 mimics | Attenuated expression of miR-185 and miR-186 may be responsible for collagen V overexpression during IPF, and these miRNAs may serve as pathogenesis-related biomarkers and treatment targets. |
| S Li et al. (2016) [23] | miR-130b-3p (↓) | Lung | IPF=4, healthy controls=3 | qPCR, miR-130b-3p mimic and inhibitor | miR-130b-3p downregulation contributed to the activation of fibroblasts and the dysregulated epithelial-mesenchymal crosstalk, suggesting its regulatory role in preventing lung fibrosis |
| H Cui et al. (2016) [24] | miR-27a-3p (↓) | Lung fibroblasts (control) and myofibroblasts (IPF) | IPF=6, healthy controls=6 | qPCR, miR-27a-3p mimic and inhibitor | This study discovered that miR-27a-3p was a negative regulator of lung myofibroblast differentiation and pulmonary fibrosis. |
| H Min et al. (2016) [25] | miR-25-3p (↑), let-7d-5p (↓) | Plasma | Acute exacerbation IPF=15, Stable-IPF=48 and healthy controls=54 | Fibrotic-associated miRNAs arrays, qPCR | A combination of miR-25-3p and let-7d-5p may be a potential biomarker for acute exacerbation IPF from IPF. |
| S Disayabutr et al. (2016) [26] | miR-34a (↑), miR-34b (↑), and miR-34c (↑) | AECs | IPF=15, healthy controls=15 | miRNA arrays, qPCR | The relative levels of senescence-associated miRNAs miR-34a, miR-34b, and miR-34c were significantly higher in AECs from IPF patients |
| X Ji et al. (2015) [27] | miR-486-5p (↓) | Lung  Serum | IPF=5, silicosis=5, healthy controls=2  silicosis=60, healthy controls=20 | qPCR | Functional test revealed that miR-486-5p may inhibit pulmonary fibrosis. |
| G Yang et al. (2015) [28] | miR-21 (↑), miR-199a-5p (↑), miR-200c (↑), miR-31 (↓), let-7a (↓), and let-7d (↓) | Serum | Profiling: Rapidly progressive IPF=12, slowly progressive IPF=12, healthy  controls=12; Validation: Rapidly progressive IPF=20, slowly progressive IPF=24, healthy  controls=20 | miRNA array, qPCR | Circulating miRNAs in serum could be potentially served as novel regulators influencing disease progression of IPF. |
| RS Nho et al. (2014) [29] | miR-96 (↑) | Lung, pulmonary fibroblasts | IPF=8, healthy controls=8 | qPCR, miR-96-5p mimic/antimir | The alteration of miR-96 expression in IPF fibroblasts contributes to maintain their pathological phenotype, which may contribute to the progression of IPF. |
| B Berschneidera et al. (2014) [30] | miR-30a (↓), miR-30d (↓), miR-92a-3p (↓), miR-338 (↓) | Lung, pulmonary fibroblasts | IPF=8, healthy controls=7 | qPCR, miR-30a-5p, 30d-5p, 92a-3p mimic/antimir | Regulatory role of miR-92a for WNT1-inducible signaling pathway protein 1 expression in pulmonary fibrosis. |
| P Li et al. (2014) [31] | miR-3675-3p (↑), miR-21 (↑), miR-155 (↑), miR-142-5p (↓), miR-557 (↓), miR-187-5p (↓), miR-101-3p (↓) | Serum | IPF=76, healthy controls=73 | Microarray, qPCR | Altered expression levels of miR-21, miR-155 and miR-101-3p were associated with FVC and radiological features in IPF. |
| V Bodempudi et al. (2014) [32] | miR-210 (↑) | Lung fibroblasts | IPF=7, healthy controls=6 | qPCR, miR-210 overexpression / inhibition | Pathological feed-forward loop exists in the IPF lung, in which hypoxia promotes IPF fibroblast proliferation via stimulation of miR-210 expression, which in turn worsens hypoxia |
| M Yamada et al. (2013) [33] | miR-21 (↑), miR-200c (↓) | Lung, AECs | IPF=3, healthy controls=3 | qPCR, miR-21 mimic | miR-21 is increased in AECs during lung fibrosis and it promotes epithelial-mesenchymal transition. |
| P Li et al. (2013) [34] | miR-21 (↑) | Serum | IPF=65, healthy controls=65 | qPCR | Serum miR-21 is associated with IPF and the degree of damage indicated by FVC and radiologic examinations. |
| CL Lino Cardenas et al. (2013) [35] | miR-199a-5p (↑) | Lung | IPF= 94, healthy controls=83 | qPCR, miR-199a-5p mimic | MiR-199a-5p behaves as a major mediator of lung fibrosis by promoting the pathogenic activation of pulmonary fibroblasts including proliferation, migration, invasion, and differentiation into myofibroblasts. |

***Abbreviations: AECs****: alveolar epithelial cells;* ***BALF****: Bronchoalveolar lavage fluid;* ***BLM:*** *bleomycin* *;* ***FFPE****: Formalin fixed paraffin embedded;* ***FVC****: Forced vital capacity;* ***GEO****: Gene Expression Omnibus;* ***IPF****: idiopathic pulmonary fibrosis;* ***PBMC****: Peripheral Blood Mononuclear Cell;* ***TAB3****: TGF‑β activated kinase 1/MAP3K7 binding protein 3.* ***↑****: high levels;* ***↓****: low levels.*

**REFERENCES:**

1. Lacedonia D, Scioscia G, Soccio P, Conese M, Catucci L, Palladino GP, et al. Downregulation of exosomal let-7d and miR-16 in idiopathic pulmonary fibrosis. BMC Pulm Med. 2021;21:188.

2. Prêle CM, Iosifidis T, McAnulty RJ, Pearce DR, Badrian B, Miles T, et al. Reduced SOCS1 Expression in Lung Fibroblasts from Patients with IPF Is Not Mediated by Promoter Methylation or Mir155. Biomedicines. 2021;9:498.

3. Sato S, Chong SG, Upagupta C, Yanagihara T, Saito T, Shimbori C, et al. Fibrotic extracellular matrix induces release of extracellular vesicles with pro-fibrotic miRNA from fibrocytes. Thorax. 2021;76:895–906.

4. Epstein Shochet G, Israeli-Shani L, Kains I, Wand O, Shitrit D. MiR-608 overexpression in idiopathic pulmonary fibrosis (IPF). BMC Pulm Med. 2021;21:1.

5. Casanova NG, Zhou T, Gonzalez-Garay ML, Lussier YA, Sweiss N, Ma S-F, et al. MicroRNA and protein-coding gene expression analysis in idiopathic pulmonary fibrosis yields novel biomarker signatures associated to survival. Transl Res. 2021;228:1–12.

6. Guiot J, Cambier M, Boeckx A, Henket M, Nivelles O, Gester F, et al. Macrophage-derived exosomes attenuate fibrosis in airway epithelial cells through delivery of antifibrotic miR-142-3p. Thorax. 2020;75:870–81.

7. Li Q, Li M, Zheng K, Li H, Yang H, Ma S, et al. Detection of microRNA expression levels based on microarray analysis for classification of idiopathic pulmonary fibrosis. Exp Ther Med. 2020;20:3096–103.

8. Kadota T, Yoshioka Y, Fujita Y, Araya J, Minagawa S, Hara H, et al. Extracellular Vesicles from Fibroblasts Induce Epithelial-Cell Senescence in Pulmonary Fibrosis. Am J Respir Cell Mol Biol. 2020;63:623–36.

9. Chen Y, Zhao X, Sun J, Su W, Zhang L, Li Y, et al. YAP1/Twist promotes fibroblast activation and lung fibrosis that conferred by miR-15a loss in IPF. Cell Death Differ. 2019;26:1832–44.

10. Njock M-S, Guiot J, Henket MA, Nivelles O, Thiry M, Dequiedt F, et al. Sputum exosomes: promising biomarkers for idiopathic pulmonary fibrosis. Thorax. 2019;74:309–12.

11. Mullenbrock S, Liu F, Szak S, Hronowski X, Gao B, Juhasz P, et al. Systems Analysis of Transcriptomic and Proteomic Profiles Identifies Novel Regulation of Fibrotic Programs by miRNAs in Pulmonary Fibrosis Fibroblasts. Genes (Basel). 2018;9:E588.

12. Liu B, Jiang T, Hu X, Liu Z, Zhao L, Liu H, et al. Downregulation of microRNA‑30a in bronchoalveolar lavage fluid from idiopathic pulmonary fibrosis patients. Mol Med Rep. 2018;18:5799–806.

13. Liu B, Li R, Zhang J, Meng C, Zhang J, Song X, et al. MicroRNA-708-3p as a potential therapeutic target via the ADAM17-GATA/STAT3 axis in idiopathic pulmonary fibrosis. Exp Mol Med. 2018;50:e465.

14. Li R, Wang Y, Song X, Sun W, Zhang J, Liu Y, et al. Potential regulatory role of circular RNA in idiopathic pulmonary fibrosis. International Journal of Molecular Medicine. 2018;42:3256–68.

15. Xie T, Liang J, Geng Y, Liu N, Kurkciyan A, Kulur V, et al. MicroRNA-29c Prevents Pulmonary Fibrosis by Regulating Epithelial Cell Renewal and Apoptosis. Am J Respir Cell Mol Biol. 2017;57:721–32.

16. Huang C, Xiao X, Yang Y, Mishra A, Liang Y, Zeng X, et al. MicroRNA-101 attenuates pulmonary fibrosis by inhibiting fibroblast proliferation and activation. J Biol Chem. 2017;292:16420–39.

17. Cui H, Ge J, Xie N, Banerjee S, Zhou Y, Antony VB, et al. miR-34a Inhibits Lung Fibrosis by Inducing Lung Fibroblast Senescence. Am J Respir Cell Mol Biol. 2017;56:168–78.

18. Shetty SK, Tiwari N, Marudamuthu AS, Puthusseri B, Bhandary YP, Fu J, et al. p53 and miR-34a Feedback Promotes Lung Epithelial Injury and Pulmonary Fibrosis. American Journal of Pathology. 2017;187:1016–34.

19. Zhang S, Liu H, Liu Y, Zhang J, Li H, Liu W, et al. miR-30a as Potential Therapeutics by Targeting TET1 through Regulation of Drp-1 Promoter Hydroxymethylation in Idiopathic Pulmonary Fibrosis. Int J Mol Sci. 2017;18:E633.

20. Tsitoura E, Wells AU, Karagiannis K, Lasithiotaki I, Vasarmidi E, Bibaki E, et al. MiR-185/AKT and miR-29a/Collagen 1a pathways are activated in IPF BAL cells. Oncotarget. 2016;7:74569–81.

21. Makiguchi T, Yamada M, Yoshioka Y, Sugiura H, Koarai A, Chiba S, et al. Serum extracellular vesicular miR-21-5p is a predictor of the prognosis in idiopathic pulmonary fibrosis. Respir Res. 2016;17:110.

22. Lei G-S, Kline HL, Lee C-H, Wilkes DS, Zhang C. Regulation of Collagen V Expression and Epithelial-Mesenchymal Transition by miR-185 and miR-186 during Idiopathic Pulmonary Fibrosis. Am J Pathol. 2016;186:2310–6.

23. Li S, Geng J, Xu X, Huang X, Leng D, Jiang D, et al. miR-130b-3p Modulates Epithelial-Mesenchymal Crosstalk in Lung Fibrosis by Targeting IGF-1. PLoS One. 2016;11:e0150418.

24. Cui H, Banerjee S, Xie N, Ge J, Liu R-M, Matalon S, et al. MicroRNA-27a-3p Is a Negative Regulator of Lung Fibrosis by Targeting Myofibroblast Differentiation. Am J Respir Cell Mol Biol. 2016;54:843–52.

25. Min H, Fan S, Song S, Zhuang Y, Li H, Wu Y, et al. Plasma microRNAs are associated with acute exacerbation in idiopathic pulmonary fibrosis. Diagn Pathol. 2016;11:135.

26. Disayabutr S, Kim EK, Cha S-I, Green G, Naikawadi RP, Jones KD, et al. miR-34 miRNAs Regulate Cellular Senescence in Type II Alveolar Epithelial Cells of Patients with Idiopathic Pulmonary Fibrosis. PLoS One. 2016;11:e0158367.

27. Ji X, Wu B, Fan J, Han R, Luo C, Wang T, et al. The Anti-fibrotic Effects and Mechanisms of MicroRNA-486-5p in Pulmonary Fibrosis. Sci Rep. 2015;5:14131.

28. Yang G, Yang L, Wang W, Wang J, Wang J, Xu Z. Discovery and validation of extracellular/circulating microRNAs during idiopathic pulmonary fibrosis disease progression. Gene. 2015;562:138–44.

29. Nho RS, Im J, Ho Y-Y, Hergert P. MicroRNA-96 inhibits FoxO3a function in IPF fibroblasts on type I collagen matrix. Am J Physiol Lung Cell Mol Physiol. 2014;307:L632-642.

30. Berschneider B, Ellwanger DC, Baarsma HA, Thiel C, Shimbori C, White ES, et al. miR-92a regulates TGF-β1-induced WISP1 expression in pulmonary fibrosis. Int J Biochem Cell Biol. 2014;53:432–41.

31. Li P, Li J, Chen T, Wang H, Chu H, Chang J, et al. Expression analysis of serum microRNAs in idiopathic pulmonary fibrosis. Int J Mol Med. 2014;33:1554–62.

32. Bodempudi V, Hergert P, Smith K, Xia H, Herrera J, Peterson M, et al. miR-210 promotes IPF fibroblast proliferation in response to hypoxia. American Journal of Physiology - Lung Cellular and Molecular Physiology. 2014;307:L283–94.

33. Yamada M, Kubo H, Ota C, Takahashi T, Tando Y, Suzuki T, et al. The increase of microRNA-21 during lung fibrosis and its contribution to epithelial-mesenchymal transition in pulmonary epithelial cells. Respir Res. 2013;14:95.

34. Li P, Zhao G-Q, Chen T-F, Chang J-X, Wang H-Q, Chen S-S, et al. Serum miR-21 and miR-155 expression in idiopathic pulmonary fibrosis. J Asthma. 2013;50:960–4.

35. Lino Cardenas CL, Henaoui IS, Courcot E, Roderburg C, Cauffiez C, Aubert S, et al. miR-199a-5p Is upregulated during fibrogenic response to tissue injury and mediates TGFbeta-induced lung fibroblast activation by targeting caveolin-1. PLoS Genet. 2013;9:e1003291.
